# Date Palm Trees Root-Derived Endophytes as Fungal Cell Factories for Diverse Bioactive Metabolites

**DOI:** 10.3390/ijms19071986

**Published:** 2018-07-07

**Authors:** Fedia Ben Mefteh, Amal Daoud, Ali Chenari Bouket, Bathini Thissera, Yamina Kadri, Hafsa Cherif-Silini, Manal Eshelli, Faizah N. Alenezi, Armelle Vallat, Tomasz Oszako, Adel Kadri, José María Ros-García, Mostafa E. Rateb, Neji Gharsallah, Lassaad Belbahri

**Affiliations:** 1 Faculty of Science, B.P. 1171, 3000, University of Sfax, 3029 Sfax, Tunisia; fedia.benmefteh@gmail.com (F.B.M.); amal.daoud42@gmail.com (A.D.); lukadel@yahoo.fr (A.K.); Neji.Gharsallah@fss.rnu.tn (N.G.); 2 Plant Protection Research Department, East Azarbaijan Agricultural and Natural Resources Research and Education Center, AREEO, 5153715898 Tabriz, Iran; ali.chenari.bouket@hotmail.com; 3 School of Science and Sport, University of the West of Scotland, Paisley PA1 2BE, UK; bathinifirst@gmail.com (B.T.); M.Eshelli@hotmail.com (M.E.); Mostafa.Rateb@uws.ac.uk (M.E.R.); 4 Labroratory of Animal Physiology, Faculty of Sciences of Sfax, University of Sfax,95, 3052 Sfax, Tunisia; Kadriamina2@gmail.com; 5 Laboratory of Applied Microbiology, Department of Microbiology, Faculty of Natural and Life Sciences, Ferhat Abbas University, 19000 Setif, Algeria; cherifhafsa@yahoo.fr; 6 Department of Food Science & Technology, Faculty of Agriculture, University of Tripoli, 13275 Tripoli, Libya; 7 NextBiotech, 98 Rue Ali Belhouane, 3030 Agareb, Tunisia; falenezi@abdn.ac.uk; 8 Neuchâtel Platform of Analytical Chemistry, Institute of Chemistry, University of Neuchâtel, 2000 Neuchâtel, Switzerland; armelle.vallat@unine.ch; 9 Forest Research Institute, 05-090 Raszyn, Poland; T.Oszako@ibles.waw.pl; 10 Department of Food Science & Technology and Human Nutrition, University of Murcia, 30100 Murcia, Spain; jmros@um.es; 11 Laboratory of Soil Biology, University of Neuchatel, 2000 Neuchatel, Switzerland

**Keywords:** brittle leaf disease, antimicrobial activity, anti-diabetic, cytotoxic, anti-obesity, anti-inflammatory, anti-haemolysis, anti-oxidant

## Abstract

Endophytic fungi of healthy and brittle leaf diseased (BLD) date palm trees (*Phoenix dactylifera* L.) represent a promising source of bioactive compounds with biomedical, industrial, and pharmaceutical applications. The fungal endophytes *Penicillium citrinum* isolate TDPEF34, and *Geotrichum candidum* isolate TDPEF20 from healthy and BLD date palm trees, respectively, proved very effective in confrontation assays against three pathogenic bacteria, including two Gram-positive bacteria *Bacillus thuringiensis* (*Bt*) and *Enterococcus faecalis* (*Ef*), and one Gram-negative bacterium *Salmonella enterica* (*St*). They also inhibited the growth of three fungi *Trichoderma* sp. (*Ti*), *Fusarium sporotrichioides* (*Fs*), *Trichoderma* sp. (*Ts*). Additionally, their volatile organic compounds (VOCs) were shown to be in part responsible for the inhibition of *Ti* and *Ts* and could account for the full inhibition of Fs. Therefore, we have explored their potential as fungal cell factories for bioactive metabolites production. Four extracts of each endophyte were prepared using different solvent polarities, ethanol (EtOH), ethyl acetate (EtOAc), hexane (Hex), and methanol (MetOH). Both endophyte species showed varying degrees of inhibition of the bacterial and fungal pathogens according to the solvent used. These results suggest a good relationship between fungal bioactivities and their produced secondary metabolites. Targeting the discovery of potential anti-diabetic, anti-hemolysis, anti-inflammatory, anti-obesity, and cytotoxic activities, endophytic extracts showed promising results. The EtOAc extract of *G. candidum* displayed IC_50_ value comparable to the positive control diclofenac sodium in the anti-inflammatory assays. Antioxidant activity was evaluated using α,α-diphenyl-β-picrylhydrazyl (DPPH), β-carotene bleaching, reducing power (RP), and 2,2-azino-bis(3-ethylbenzothiazoline-6-sulphonique) (ABTS) radical scavenging assays. The findings revealed strong anti-oxidant power with an IC_50_ of 177.55 µg/mL for *G. candidum* EtOAc extract using DPPH assay, probably due to high polyphenol and flavonoid content in both fungal extracts. Finally, LC-HRMS (Liquid Chromatography–High Resolution Mass Spectrometry) and GC-MS (Gas Chromatography–Mass Spectrometry) analysis of *G. candidum* and *P. citrinum* extracts revealed an impressive arsenal of compounds with previously reported biological activities, partly explaining the obtained results. Finally, LC-HRMS analysis indicated the presence of new fungal metabolites that have never been reported, which represent good candidates to follow for the discovery of new bioactive molecules.

## 1. Introduction

Fungi are known to inhabit almost all ecological niches on Earth, in addition to their ability to produce diverse secondary metabolites [1]. Given their extreme variability in chemical structure, as well as bioactive properties, fungal secondary metabolites are precious natural compounds. Therefore, considerable biotechnological and economic interest allowed the build-up of specific, fast-growing, and dedicated research communities [2,3]. Numerous studies found a strong anti-oxidant power in fungal extracts correlated with their total phenolic contents [3,4]. Antioxidants, acting as radical-scavengers and inhibiting harmful free radical-mediated processes, such as lipid peroxidation, are believed to protect human cells against free radicals generated during numerous diseases [5]. Fungal extracts have also been shown to be endowed with many biological activities including anti-inflammatory [6], anti-haemolysis [6], anti-diabetic [7], anti-obesity [8], anti-proliferative [9], and antimicrobial activities [1]. Additionally, their volatile organic compounds (VOCs) also proved efficient as antibacterial agents [1].

Fungal endophytes, like those that reside inside plants, but do not cause any deleterious effect to them, are responsible for different biological activities of their host plant, but their actual role is still under investigation [1,10]. Therefore, endophyte communities are being characterized from plants and known for their diverse biological activities [1,11]. Additionally, fungal endophytes are recognized as alternative source of their hosts for the production of volatile organic compounds (VOCs) [12], rich resource of antibiotics [13], and other biologically-active natural products [14].

Emerging analytical hyphenated techniques, such as GC-MS and LC-MS analyses, are being widely applied for dereplication of bioactive fungal secondary metabolites [15]. Additionally, large-scale fermentation and purification of bioactive products is used to characterise endophytic natural products diversity and link them to their biological activity [1]. Despite the importance of mining of secretomes and volatilomes of microbes, in general, and plant-associated microbes, few studies have been dedicated [16,17].

Recently, fungal cell factories are attracting significant interest due to their biotechnological potential [1,18,19,20]. The strategy has been widely applied to primary metabolism towards the production of enzymes or their derived metabolites. Being applied to primary metabolism, fungal cell factories were mainly applied to model experimentally-tractable systems [21]. Numerous studies focused on characterization of secondary metabolites and their encoding clusters in experimentally-tractable model species [22]. However, many non-experimentally tractable fungi are either known to produce valuable secondary metabolites or require specific conditions to accumulate them. The advent of new state-of-art low-cost genomic sequencing techniques open new opportunities to identify secondary metabolite gene inventories in fungal endophytes [1]. Rapid genome-wide identification, annotation, and analysis of secondary metabolite biosynthesis gene clusters in a given genome is possible nowadays. For instance, secondary metabolite gene cluster analysis using antiSMASH 3.0 [23], prediction informatics for secondary metabolomes (PRISM) [24], NapDos [25], NP.search [26], and the bacteriocin specific software BAGEL3 [27] allowed the identification of numerous secondary metabolite biosynthetic gene clusters that are useful to perform genome mining. Mining of available genomes and the expression of unknown clusters relevant to secondary metabolites in heterologous systems is a promising strategy that already allowed the discovery of a large number of new metabolites [28].

In the current study, we used two non-experimentally tractable fungi namely *Penicillium citrinum* TDPEF34 and *Geotrichum candidum* TDPEF20 originating from healthy and BLD date palm root microbiomes, respectively, towards harnessing them for use as cell factories for bioactive secondary metabolites production [1]. Extracts of both endophytic fungi proved polyphenol- and flavonoid-rich and, as a consequence, exhibited strong antioxidant power. Fungal extracts were also endowed with highly valuable anti-haemolytic, anti-inflammatory, anti-diabetic, anti-obesity, anti-proliferative, and antimicrobial activities. LC-MS and GC-MS analyses allowed the discovery of numerous secondary metabolites and VOCs with known biological activities and provided a list of unknown secondary metabolites as putative candidates for the discovery of new bioactive natural products. Mining of the genomes of the two-species allowed us to describe secondary metabolite biosynthetic gene clusters. Cross-talk between chemical analysis and genome sequencing studies as well as the input of phylogenomics in the field are discussed in this report.

## 2. Results

### 2.1. Antimicrobial (Antagonistic) Activities of G. candidum and P. citrinum and Their Volatiles

**Figure 1:**
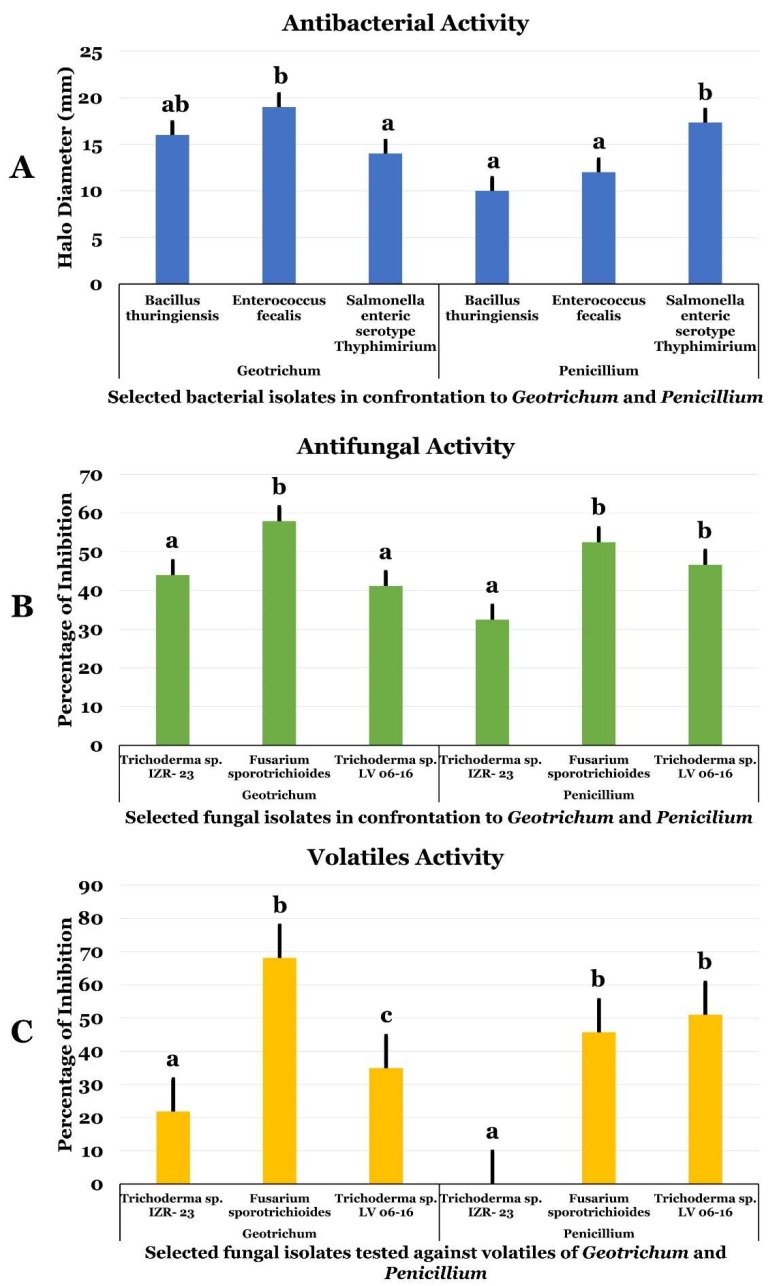
Antibacterial (**A**), antifungal (**B**), and volatile (**C**) activities of examined *Geotrichum* and *Penicillium* (Hex, EtOAc, MetOH, EtOH) against pathogenic bacteria and fungi. Data presents the mean ± standard error. Bars labelled with different letters are significantly different among the treatments at *p* < 0.05 using the Tukey’s HSD test. In each bar groups, bars labelled with the same letter are not significantly different from each other according to Tukey’s HSD at *p* < 0.05.

*Geotrichum candidum* and *Penicillium citrinum* were examined against several bacteria and phytopathogen fungi to assess their antimicrobial activities (Figure 1). Both endophytic fungi inhibited the three-bacterial species tested *Bacillus thuringiensis* (*Bt*), *Enterococcus faecalis* (*Ef*), and *Salmonella enterica* subsp. *enterica* serovar Typhimurium (*St*) (Figure 1A). The average diameter of the inhibition halo was in the range of 10 to 19 mm. The highest halo diameter was recorded against *Ef* with *G. candidum*. *Penicillium citrinum* showed the highest inhibition efficiencies against *St* (Figure 1A). The endophyte *Geotrichum* showed stronger inhibition of Gram-positive bacteria *Ef* and *Bt.* The pathogen *Salmonella enterica* subsp. *enterica* serovar Typhimurium (*St*) was the most sensitive when confronted with date palm endophytes.

Regarding antifungal activity, the percentage of inhibition was between 32.4% and 57.9%. As shown in Figure 1B, the highest percentage of inhibition was obtained with *G. candidum* against *F. sporotroichoides* (*Fs*)*.* Both *G. candidum* and *P. citrinum* were more effective against phytopathogenic fungi than bacteria (Figure 1B). Interestingly, *Fs* was more sensitive than the other tested phytopathogenic fungi with percentage of inhibition higher than 50%.

Volatile organic compounds (VOCs) from both *G. candidum* and *P. citrinum* were assessed by double culture method. The inhibition percentage was calculated and is presented in Figure 1C. The findings revealed that these endophytic strains were able to produce antifungal volatile compounds. However, the inhibition percentage recorded ranged between 21.85% and 68.09%. Noteworthy, VOCs of *G. candidum* significantly inhibited *Fs*. VOCs of *G. candidum* accounted for half and total inhibition of *Ti* and *Ts*, respectively. However, VOCs of *P. citrinum* exhibited no effect against *Ti*, but accounted for the full inhibition against *Ts* (Figure 1C).

### 2.2. Antimicrobial Assay of G. candidum and P. citrinum Extracts

**Figure 2:**
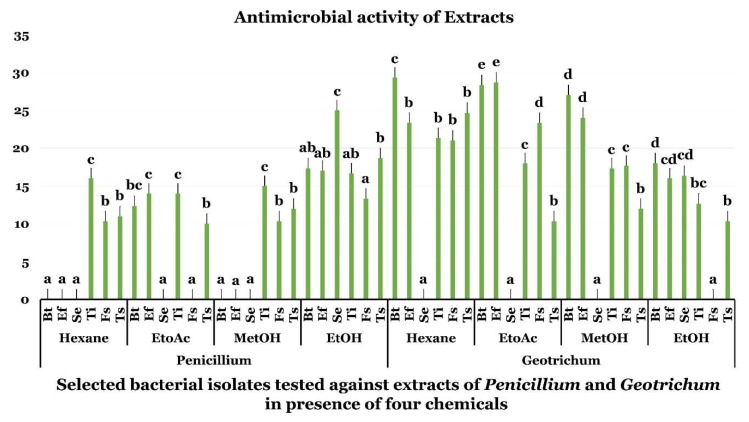
Antimicrobial activity of extracts of *Geotrichum* and *Penicillium* against bacterial and fungal species in the presence of hexane, EtOAc, MetOH, EtOH. Data presents mean ± standard error. Bars labelled with different letters are significantly different among the treatments at *p* < 0.05 using the Tukey’s HSD test. In each bar groups, bars labelled with the same letter are not significantly different from each other according to Tukey’s HSD at *p* < 0.05.

Antimicrobial activities of *G. candidum* and *P. citrinum* extracts have been accessed in vitro against three bacterial and three fungal pathogens using agar diffusion well method (Figure 2). Both fungal species showed varying degrees of inhibition of the bacterial and fungal pathogens according to the solvent used. The highest inhibition diameter was obtained with hexane extract of *G. candidum* against *Bacillus thuringiensis* (*Bt*). However, EtOH proved effective in extracting *P. citrinum* broad range bioactive compounds from *P. citrinum* that inhibited the tested bacterial and fungal pathogens (Figure 2). Hexane, EtOAc, and MetOH proved effective in recovering highly active fractions from *G. candidum* that proved active against most of the tested pathogens (Figure 2).

### 2.3. Biological Activities of G. candidum and P. citrinum Extracts

Both *G. candidum* and *P. citrinum* displayed interesting biological activities in vitro (Table 1). *G. candidum* have higher anti-diabetic, anti-inflammatory, anti-obesity, and cytotoxic activities when using EtOAc extracts. Better anti-haemolytic activity was, however, obtained using Hex extract (Table 1). IC_50_ values displayed by the extracts were 280 (cytotoxic), 12.5 (anti-obesity), 6.6 (anti-diabetic), 2.8 (Anti-haemolysis), and 1.2 (anti-inflammatory) times higher than values obtained with the positive control drugs paclitaxel, orlistat, acarbose, aspirin, and diclofenac sodium, respectively. *P. citrinum* displayed the best anti-diabetic and anti-haemolytic activities using MetOH, anti-inflammatory activity using EtOH and anti-obesity activity using EtOAc extracts (Table 1). It is worth mentioning that *P. citrinum* extracts did not display any cytotoxic activity against HepG2 cells using all extract types (Table 2). IC_50_ values displayed by the extracts were 13.5 (anti-obesity), 9.7 (anti-diabetic), and 4.6 (anti-haemolytic and anti-inflammatory) times higher than positive control drugs orlistat, acarbose, aspirin, and diclofenac sodium, respectively (Table 1).

### 2.4. Chemical Composition of G. candidum and P. citrinum Extracts

#### Polyphenol and Flavonoid Contents

Extraction of *G. candidum* and *P. citrinum* using the different solvents allowed the recovery of fractions with different total polyphenols and flavonoid contents (Table 2). The polyphenol content of fungal extracts was between 16.69 ± 1.51 and 33.39 ± 0.49 mg GAE/g. *Geotrichum candidum* possessed higher polyphenol contents than *P. citrinum*. For *G. candidum*, EtOAc was the most effective solvent in extracting polyphenols, while for *P. citrinum*, MetOH was the most efficient (Supplementary Figure S1). *Geotrichum candidum* exhibited the highest flavonoid contents recorded with the solvent EtOH (14.41 ± 0.006 mg QE/g). For *P. citrinum*, EtOH and MetOH were both more effective in extracting flavonoids. Therefore, polar solvents give higher yields of polyphenol and flavonoids for *P. citrinum*. For *G. candidum*, medium polarity solvent was more suitable to maximize polyphenol extraction and a polar solvent for better yield of flavonoids (Supplementary Figure S1).

### 2.5. In Vitro Antioxidant Activity of Date Palm Endophytic Fungi

Antioxidant activity of *P. citrinum* and *G. candidum* were investigated by four complementary tests listed below:

#### 2.5.1. DPPH Free Radical-Scavenging Activity

DPPH free radical scavenging assay was studied for *G. candidum* and *P. citrinum* extracts. This test evaluates the ability of an antioxidant to inhibit oxidative cell damages preventing the attack of key biomolecules by the reactive radical species. Results are presented in Table 2 as IC_50_ values. The lowest IC_50_ value would reflect the best antioxidant effect of the sample. The results clearly indicate that *Geotrichum* extracts show higher antioxidant activity than *Penicillium* extracts. Among all extracts, the EtOAC extract exhibited the highest scavenging activity (IC_50_ = 177.55 ± 0.96 µg/mL) followed by the MetOH extract of *Geotrichum* (IC_50_ = 207.44 ± 1.48 µg/mL). Regarding *Penicillium* extracts, the best antioxidant activity was obtained with MetOH (IC_50_ = 227.87 ± 0.49 µg/mL). The differences observed in radical scavenging effect between *Geotrichum* and *Penicillium* extracts could be attributed to the differences in its polyphenol content.

#### 2.5.2. β-Carotene-Linoleic Acid Assay

The antioxidant potential of the endophytic fungi extracts was assessed using β-carotene bleaching test based on their ability to inhibit the peroxidation of linoleic acid. The concentrations displaying 50% inhibition were expressed as IC_50_ values. *Geotrichum* and *Penicillium* extracts were able to inhibit lipid peroxidation (Table 2). EtOAc extract of *Geotrichum* displayed the highest level of antioxidant activity (IC_50_ = 162.86 ± 0.34 µg/mL), compared to BHT used as positive control (IC_50_ = 50.11 ± 0.18 µg/mL). IC_50_ values of *Penicillium* extracts were in the range of 235.28 ± 1.15–264.55 ± 2.59 µg/mL showing lower antioxidant activity in comparison with *Geotrichum* samples.

#### 2.5.3. Reducing Power Assay

The ability of *Geotrichum* and *Penicillium* extracts to reduce Fe^3+^/Ferricyanide complex to its ferrous form was evaluated. As shown in Table 2, the reducing power of the fungal samples seemed to be dependent on their total phenolic and total flavonoid content.

*Geotrichum candidum* EtOAc extracts displayed the highest reduction level recorded (IC_50_ = 190.3 ± 3.36 µg/mL), reflecting its antioxidant potential. The IC_50_ value of the ascorbic acid used as the positive control was 182.08 ± 1.12 µg/mL. For *P. citrinum* samples, MetOH extract exhibited the highest rate of reduction activity (IC_50_ = 233.89 ± 3.32 µg/mL) followed by EtOAc extract (IC_50_ = 264.76 ± 4.43 µg/mL).

#### 2.5.4. ABTS Radical-Scavenging Activity

Radical scavenging activity of *Geotrichum* and *Penicillium* extracts was investigated using ABTS assay (Table 2). Two positive controls were used, namely Trolox and ascorbic acid. The findings revealed higher antioxidant activity of *G. candidum* than *P. citrinum* extracts. EtOAc sample possessed the highest ABTS scavenging potential with IC_50_ value of 151.31 ± 4.2 µg/mL. *Geotrichum* extracts had the highest antioxidant rate according to the following order: EtOAc > MetOH > EtOH > Hex. The antioxidant potential of *Penicillium citrinum* extracts varied following the order: MetOH > EtOAc > EtOH > Hex. It was worth noticing that hexane solvent displayed the lowest antioxidant rate for both *Penicillium* and *Geotrichum* fungi. The antioxidant potential varies with the polarity of the solvent.

### 2.6. LC-HRMS Analysis of G. candidum and P. citrinum Extracts

Analysis of *G. candidum* extracts by LC-HRMS proved effective in detecting bioactive compounds that have been reported for their antimicrobial activity such as clavicipitic acid, 7-butyl-6,8-dihydroxy-3-pent-11-enylisochromen-1-one, cyclo(L-Leu-L-Pro), cyclo(L-Tyr-L-Pro), cyclo-(L-Pro-L-Val), 8-methoxytrypethelone methyl ether, brasilamide F, cordiachrome D, Sch-725674, carbonarin E, cytochalasin J, β-hydroxy mevinolin, g-hydroxy mevinolin, cyclo(Phenylalanyl-n-methyltyrosyl), 14-aza-24-methylene-d-homocholesta-8,14-dien-3-ol,acetoxy-24-methylene-14a-aza-n-homo-5a cholesta-8,14-diene, 3-Keto-24-methylene-14a-aza-d-homo-5a-cholesta-8,14-diene, and 4a-methyl-15-aza-24-methylene-d-homocholesta-8,14-dien-3-ol (Supplementary Table S1). Additionally, other identified compounds have been reported to possess anti-diabetic and anti-haemolytic activities (cytochalasin J), anti-inflammatory activity (cyclo(L-Leu-L-Pro), cyclo(L-Tyr-L-Pro), cyclo-(L-Pro-L-Val), cytochalasin J), anti-obesity activity (β-hydroxy mevinolin and g-hydroxy mevinolin), and cytotoxic activity such as clavicipitic acid, 7-butyl-6,8-dihydroxy-3-pent-11-enylisochromen-1-one, cyclo(L-Leu-L-Pro), cyclo(L-Tyr-L-Pro), cyclo-(L-Pro-L-Val), 8-methoxytrypethelone methyl ether, 7-methoxy-4,8,9-trihydroxy-1,6,7,8-tetrahydro-2*H*-benzo[*j*]fluoranthen-3-one, brasilamide F, cordiachrome D, carbonarin E, virescenoside E, cytochalasin J, β-hydroxy mevinolin and g-hydroxy mevinolin (Supplementary Table S1). Finally, 2-methylenecycloheptene-1,3-diglycine and 4,4-dimethyl-3-hydroxy-24-methylene-14a-aza-d-homo-5a-cholesta-8,14-diene have never been reported to possess any of the tested activities.

*Penicillium citrinum* extracts analysed by LC-HRMS proved rich in compounds that were reported for their antimicrobial activity such as 1,2-dihydroxyindolizidine, cyclo(L-Pro-L-Val), cyclo(L-Tyr-L-Pro), cyclo(L-Leu-L-Pro), cyclo(L-Phe-L-Pro), lumichrome, cyclo(L-Val-L-Phe), chaetominedione, merulinic acid B, amicycline, citrinin, and 5-hydroxyvertinolide (Supplementary Table S2). Moreover, 2-isopropyl-6-methylpyrazine, cyclo(L-Pro-L-Val), cyclo(L-Tyr-L-Pro), cyclo(L-Leu-L-Pro), citrinin and 5-hydroxyvertinolide were identified in the fungal extract and were proved active against inflammation. Finally, cyclo(L-Pro-L-Val), cyclo(L-Tyr-L-Pro), cyclo(L-Leu-L-Pro), chaetominedione and citrinin, identified in the fungal extract, were reported for their cytotoxic effect (Supplementary Table S2). Cyclo(L-ala-L-Leu) and L-leucine anhydride have never been reported to display any of the biological activities tested in this study (Supplementary Table S2).

### 2.7. GC-MS Analysis of G. candidum and P. citrinum Extracts 

*Penicillium citrinum* extracts analysed using GC-MS revealed the presence of numerous compounds that possessed antimicrobial activity, such as α-pinene, camphene, phenylethanol, citronellal, terpinen-4-ol, and cuminaldehyde, anti-inflammatory activity, such as α-pinene, camphene, arginine, terpinen-4-ol and cuminaldehyde, cytotoxic effect, such as camphene and cuminaldehyde, anti-haemolytic effect, as arginine, and anti-obesity activity, such as α-pinene and camphene. All remaining VOCs detected in *P. citrinum* (Supplementary Table S3) have never been reported to possess any of the biological activities accessed in this study.

Analysis of *G. candidum* extracts by GC-MS indicated the presence of some bioactive VOCs that have been reported to exert antimicrobial activity such as cyclo(Leu-Pro), cyclo(Phe-Pro), and β-carboline. The GC-MS analysis also detected other VOCs endowed with cytotoxic activity such as ascaridole, cyclo(Leu-Pro), and β-carboline, anti-hemolytic activity as β-carboline, and anti-inflammatory activity such as cyclo(Gly-Pro) and cyclo(Leu-Pro). All the remaining VOCs detected in *G. candidum* (Supplementary Table S4) have never been reported to possess any of the biological activities targeted in the study.

### 2.8. Genome Mining of G. candidum and P. citrinum Strains

**Figure 3:**
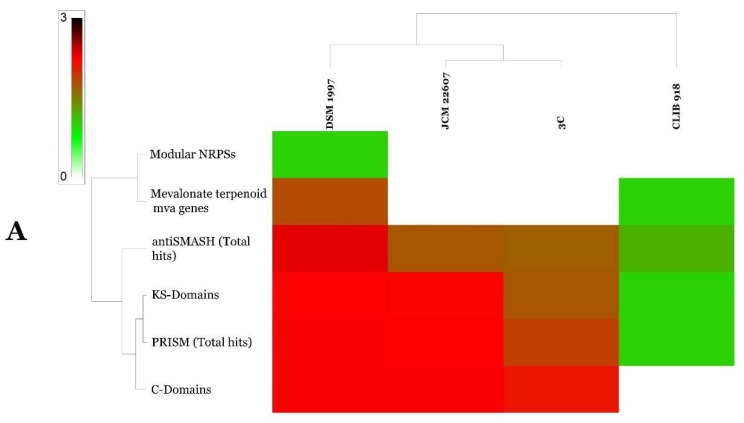

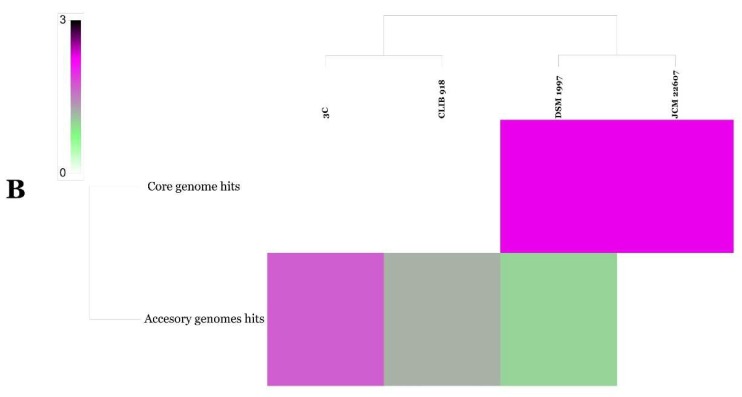
Heat map of the mining of genes contributing to secondary metabolite clusters (**A**). antiSMASH hits of core and accessory genomes (**B**) of examined *Geotrichum* and *Penicillium* strains.

GGDC analysis of both *G. candidum* and *P. citrinum genomes* allowed us to infer that *P. citrinum* strains JCM22607 (GenBank Accession: BCKA00000000.1) and DSM1997 (GenBank Accession: LKUP00000000.1) belonged to the same species and that *G. candidum* strains CLIB 918 (GenBank Accession: CCBN000000000.1) and 3C (GenBank Accession: JMRO00000000.2) were genetically very distant (Table 3). The genomes of *G. candidum* and *P. citrinum* have also been screened for the presence of secondary metabolite clusters using antiSMASH 3.0 [23], prediction informatics for secondary metabolomes (PRISM) [24], NapDos [25] and NP.search [26]. As shown in Figure 2 and Supplementary Table S2, different strains showed high levels of diverse secondary metabolite clusters using all implied programs. *P. citrinum* DSM 1997 proved very rich in secondary metabolite clusters with a total of 29 putative clusters whereas *P. citrinum* isolate JCM 22607 showed only nine clusters (Figure 3 and Supplementary Table S5). *G. candidum* isolate CLIB 918 and 3C showed two and eight clusters, respectively (Figure 3 and Supplementary Table S5). None of the biosynthetic gene clusters discovered in the four genomes showed significant similarity to known secondary metabolites, suggesting the potential for new natural products discovery (Supplementary Table S5).

## 3. Discussion

Endophytic fungi, in addition to their plant growth promoting traits, such as biological nitrogen fixation, indole-3-acetic acid biosynthesis, phosphate solubilisation, and siderophore production, have become the focus of numerous studies that acknowledge their wide antibacterial and antifungal activities with significant biotechnological interest [1]. Endophytic fungi proved effective in inhibiting emerging plant pests and pathogens [29,30]. They could be described as a treasure house of bioactive compounds, acting as reservoirs of novel bioactive secondary metabolites of relevance for anti-cancer, anti-diabetic and anti-obesity activities [31,32,33]. In the present report, we investigated the potential of two endophytic fungi from healthy and brittle leaf diseased (BLD) date palm trees (*Phoenix dactylifera* L.) for their antibacterial and antifungal activities as well as their potential as a source of bioactive compounds with industrial and pharmaceutical applications. The endophyte of healthy date palm trees, *P. citrinum* isolate TDPEF34 and of that BLD date palm trees, *G. candidum* TDPEF20 proved effective in confrontation assays against three pathogenic bacteria, including two Gram-positive bacteria *B. thuringiensis* (*Bt*) and *E. faecalis* (*Ef*) and one Gram-negative bacterium *S. enterica* subsp. *enterica* serovar Typhimurium (*St*). These results highlight their potential for effective practical application in coping with these bacteria that became more resistant to common antibiotic treatments [9]. These isolates proved also effective in inhibiting three fungi namely *Trichoderma* sp. IZR- 23 (*Ti*), *Fusarium sporotrichioides* (*Fs*), *Trichoderma* sp. LV 06-16 (*Ts*). Their VOCs were shown to be in part responsible for the inhibition of *Ti* and *Ts* and could account for the full inhibition of *Fs*. This finding is of imminent relevance for biocontrol of mycotoxin-producing fungi *Fusarium* spp., a serious threat to human and animal health which may steer to the development of biocontrol agents for the control of fungal diseases and mycotoxin production [34]. Given the results obtained using living fungal cultures, we explored the potential of these two isolates as fungal cell factories for bioactive metabolites production. Four different extracts of each endophyte with solvents of different polarities have then been prepared, ethanol (EtOH), ethyl acetate (EtOAc), hexane (Hex), and methanol (MetOH). Both endophytic species showed varying degrees of inhibition of the bacterial and fungal pathogens according to the solvent used. These results suggested a strong link between fungal bioactivities and their secondary metabolite profiles extracted using the different solvents. This result was in agreement with the results reported by Mefteh et al. [1] and Gos et al. [35]. In biological assays targeting discovery of anti-diabetic, anti-haemolytic, anti-inflammatory, anti-obesity and cytotoxic activities, all endophytic extracts showed promising results. IC_50_ values displayed by EtOAc extracts of *G. candidum* in the anti-inflammatory assays were close (IC_50_ = 0.47 ± 0.006 mg/mL) to values obtained with the positive control drug diclofenac sodium (IC_50_ = 0.41 ± 0.01 mg/mL). This result highlights the potential of EtOAc extracts of *G. candidum* to yield specific bioactive metabolite(s) that could potentially be evaluated as new anti-inflammatory drug. Anti-diabetic, anti-obesity, anti-haemolytic, and cytotoxic activities of the different extracts using the various solvents were also noticed but were weaker compared to positive control drugs used in these assays. This result could be explained by the presence of compounds that antagonize active components [36]. Therefore, we decided to proceed to large-scale fermentation, isolation, and detailed characterization of fungal extracts secondary metabolites as a future step. Both fungal endophytes proved polyphenol and flavonoid rich. Antioxidant activity was evaluated using α,α-diphenyl-β-picrylhydrazyl (DPPH), β-carotene bleaching, ferric reducing ability of plasma (FRAP), and 2,2-azino-bis(3-ethylbenzothiazoline-6-sulphonique) (ABTS) radical cation decolourization assays revealed strong anti-oxidant power with an IC_50_ of 177.55 µg/mL for *G. candidum* EtOAc extract using DPPH assay (IC_50_ of 34.33 for the positive control butylated hydroxytoluene). This result prompted us to tentatively characterize fungal extract metabolites using high-resolution LC-MS analysis [37]. LC-MS analysis revealed the presence of numerous metabolites that were reported to have antimicrobial [38,39], anti-diabetic [40], anti-haemolytic [41], anti-inflammatory [42,43], anti-obesity [44], and cytotoxic activities [45] in both fungi. GC-MS analysis also proved effective in recovering already characterized VOCs with antimicrobial [46], anti-haemolytic [42], anti-inflammatory [47], anti-obesity [48] and cytotoxic activities [49]. In addition to these metabolites that could partially account for the fungal extracts’ biological activities, LC-MS and GC-MS analyses also confirmed the presence of numerous other compounds that have never been reported to possess any of these tested activities. These compounds could be interesting candidates in new screening activities to recover fungal secondary products with biotechnological prone biological activities. Genome mining of *G. candidum* and *P. citrinum* strains for secondary metabolites biosynthesis revealed fungal strain specificity for the presence of secondary clusters (29 putative clusters for *P. citrinum* DSM 1997 strain versus nine clusters for *P. citrinum* isolate JCM 22607).

We speculate that these results showed similar trends with what have been observed for bacterial secondary metabolism [16]. However, wider sampling of *P. citrinum* strains is needed to confirm such findings. GGDC analysis of the two strains of *G. candidum* proved that they were genetically distant if species boundaries are applied sensu Meier-Kolthoff et al. [50]. Therefore, critical assessment of strains identity is required before studying its secondary metabolites as suggested by Belbahri et al. [16]. In conclusion, (i) date palm endophytic extracts represent a promising source of bioactive compounds with industrial and pharmaceutical applications; (ii) combination of biological, chemical and genomic analyses is a promising approach for the discovery of new bioactive products; and (iii) considerable taxonomic efforts should be deployed before screening and identifying fungal secondary metabolites. Our strategy proved successful and could provide an example to follow to unravel the biotechnological potential of secondary metabolites in filamentous fungi. Hence, such fungi would be considered as unique organisms in producing these secondary metabolites, which was revealed only by actinomycetes and plants [51].

## 4. Materials and Methods

### 4.1. Antimicrobial Screening G. candidum and P. citrinum Fungi and Their Extracts

In all antagonistic assays three independent experiments have been performed (biological repetitions) with three technical repetitions for each biological repetition.

#### 4.1.1. Microbial Strains and Growth Conditions

The antibacterial activity of date palm endophytic fungi extracts was assessed against three pathogenic bacteria, including two Gram-positive bacteria *Bacillus thuringiensis* (*Bt*) and *Enterococcus faecalis* (*Ef*) and one Gram-negative bacterium *Salmonella enterica* subsp. *enterica* serovar Typhimurium (*St*). Antifungal activity was performed against three phytopathogenic fungi namely *Trichoderma* sp. IZR- 23 (*Ti*), *Fusarium sporotrichioides* (*Fs*), *Trichoderma* sp. LV 06-16 (*Ts*).

#### 4.1.2. Antagonistic Assay of *G. candidum* and *P. citrinum* Fungi

The endophytic fungi of date palm *P. citrinum* TDPEF34 and *G. candidum* TDPEF20 were screened for their antimicrobial activity against three bacteria and three phytopathogenic fungi. The antibacterial activity was assessed following the protocol described by Mefteh et al. [1]. Bacterial cultures were prepared in 5 mL Mueller Hinton Broth (MHB, Sigma-Aldrich, Buchs, Switzerland) and incubated in shaker (200 rpm) at 37 °C, except for *Bacillus thuringiensis*, which was incubated at 30 °C. The optical density of overnight cultures was determined at 625 nm and adjusted from 0.08–0.10 equivalent to 107 CFU/mL, corresponding to the exponential stage of bacterial growth [1]. For antibacterial assay, the plates containing Mueller Hinton Agar (MHA, Sigma-Aldrich, Buchs, Switzerland) were inoculated with 100 µL of bacterial cultures using a sterile cotton swab (Thomas Scientific, CA, USA). Then, plugs (5 × 5 mm) from pure cultures of endophytic fungus cut with a sterile scalpel (Sigma-Aldrich, Buchs, Switzerland) and were transferred onto the surface of MHA plates. Plates were incubated for 24 h at 30 °C and the diameters of inhibition zones were recorded. Concerning antifungal activity, a dual culture method was performed following Alenezi et al. [52]. On potato dextrose agar plates (PDA), plugs from cultures of the two partners, endophytic fungus and phytopathogenic fungus, were placed with a 50 mm distance from each other in a plate of 90 mm. The control plates were inoculated only with phytopathogenic fungus. After incubation of plates at 30 °C for 72 h, the percentage of inhibition (IP) of each endophytic fungus was calculated as the following formula (A: radial diameter of phytopathogenic fungus colony growth in control plates; B: radial diameter of phytopathogen fungus colony growth in test plates):

$$\mathrm{IP}\;\left(\%\right)=\frac{\left(A-B\right)}{A}\times 100$$

#### 4.1.3. Antagonistic Assay of *G. candidum* and *P. citrinum* Volatiles

Date palm-derived endophytic fungal VOCs were screened for their antifungal potential in vitro [53]. A simple bioassay was conducted following the protocol described by Schalchli et al. [54]. Initially, an agar strip of 2.5 cm wide was completely removed from the middle of fresh PDA plates. Then, plugs from the cultures of the endophytic fungus and the phytopathogenic fungi were placed on each side of the plate. The control plates were inoculated only with examined fungi. Afterwards, the plates were sealed and incubated for 72 h at 30 °C. The potential of endophytic fungi VOCs to inhibit the growth of phytopathogenic fungi was evaluated by determination of percentage of inhibition of their radial growth (PI):

$$\mathrm{PI}\;\left(\%\right)=\frac{\left(A-B\right)}{A}\times 100$$

where A: radial diameter of phytopathogenic fungus colony growth in control plates, and B: radial diameter of phytopathogenic fungus colony growth in test plates.

#### 4.1.4. Fermentation and Preparation of Extracts of *G. candidum* and *P. citrinum*

The endophytic fungi *P. citrinum* TDPEF34 and *G. candidum* TDPEF20 were isolated as described previously from the roots of healthy and Leaf Brittle-diseased (LBD) date palm, respectively [1]. Briefly, mycelia from pure cultures of endophytic fungi were inoculated into potato dextrose broth (PDB) medium in Erlenmeyer flasks (Thermo Fisher Scientific, Foster City, CA, USA) and incubated in the shaker-incubator for 3 weeks at 30 °C, 150 rpm. Subsequently, fungal cultures were filtered through Whatman no.1 filter paper (Thomas Scientific, Foster City, CA, USA) and the resulted filtrates were extracted with *n*-hexane, ethyl acetate (EtOAc) and methanol (MetOH). The extraction was performed trice and the extracts were dried by a rotary evaporator under vacuum at 45 °C and freezing dried to complete dryness. Mycelia of endophytic fungi cultures were rinsed in sterile water and dried in an oven at 60 °C for 48 h. Then, the mycelia were ultrasonically extracted for 30 min in absolute ethanol and extracted in a similar way. The obtained extracts were stored at 4 °C before being used for further study.

#### 4.1.5. Antimicrobial Activity of *G. candidum* and *P. citrinum* Extracts

Fungal extracts were initially prepared for antimicrobial assay. The extracts were dissolved in double distilled water in order to obtain final concentration of 100 µg/mL and filtered using 0.2 µm membrane filter. Antimicrobial activity was evaluated using handmade agar-well diffusion method [1]. Briefly, 100 µL of bacterial culture or spore suspension of phytopathogenic fungi were spread on periphery of MHA and PDA plates, respectively. Afterwards, each well was filled with 60 µL of fungal extracts. The plates were incubated at 30 °C for 24 h for bacteria and for 72 h for fungi. The antimicrobial activity of fungal extracts was expressed on diameter of inhibition zones (mm). For each extract three independent experiments have been performed (biological repetitions) with three technical repetitions for each biological repetition.

### 4.2. In Vitro Biological Activities of G. candidum and P. citrinum Extracts

For each biological activity three independent experiments have been performed (biological repetitions) with three technical repetitions for each biological repetition.

#### 4.2.1. Cytotoxic Activity

The cytotoxicity of date palm fungal extracts was evaluated against the human hepatocyte carcinoma HepG2 cells using 3-(4,5-dimethyl-2-thiazolyl)-2,5-diphenyl-2*H*-tetrazolium bromide (MTT) cell viability method [55]. Briefly, HepG2 cells were cultured in MEM supplemented with 10% (*v*/*v*) foetal bovine serum, 100 U/mL penicillin, 100 µg/mL streptomycin, 1% non-essential amino acids, and 2 mM l-glutamine, in CO_2_ incubator at 37 °C with 95% humidity, and 5% CO_2_ gas environment. Afterwards, HepG2 cells (10^5^ cells/well) were seeded onto sterile 96 well plates and allowed to adhere for 24 h. Then, cells were treated with fungal extracts (25, 50, 100, 150, 200 µg/mL) and the plates were incubated for 72 h. MTT solution (Sigma-Aldrich, Buchs, Switzerland) (5 mg/mL) was then added to each well. After 4 h incubation, the supernatant was aspired and MTT-formazan precipitate was dissolved in 100 µL dimethyl sulfoxide (DMSO) (Sigma-Aldrich, Buchs, Switzerland). The absorbance was measured in a micro-plate reader (Thermo Electron Corporation, Barcelona, Spain) at a wavelength of 570 nm with a reference wavelength of 690 nm. Each sample was examined at least in triplicate per experiment. Cells treated with DMSO were used as negative control while paclitaxel was used as positive control. The results were expressed as the percentage of cells viability calculated by the following formula:

$$\mathrm{Inhibitory}\;\mathrm{ratio}\;\left(\%\right)=\frac{\left(\mathrm{OD}\;\mathrm{control}-\mathrm{OD}\;\mathrm{treated}\right)}{\mathrm{OD}\;\mathrm{control}}\times 100$$

where OD refers to optical density.

#### 4.2.2. Anti-Inflammatory Activity

Anti-inflammatory activity of these endophytic extracts was evaluated by protein denaturation method [56]. Briefly, different concentrations of fungal extracts (0.25–2 mg/mL) were mixed with 500 µL of 5% bovine serum albumin. The mixtures were left standing at room temperature for 15 min. Then, protein denaturation was induced by heating the reaction mixtures at 70 °C for 20 min. After cooling, the absorbance of the solutions was measured at 660 nm. Diclofenac sodium was used as the positive control. All experiments were carried out at least in triplicate. The inhibition percentage of protein denaturation was determined using the following formula:

$$\mathrm{IP}\;\left(\%\right)=\frac{\left(\mathrm{OD}\;\mathrm{sample}-\mathrm{OD}\;\mathrm{control}\right)}{\mathrm{OD}\;\mathrm{control}}\times 100$$

where OD refers to optical density.

#### 4.2.3. Anti-Haemolytic Activity

The in vitro anti-haemolytic activity of the fungal extracts was evaluated by the method described by Hasnat et al. [57]. Erythrocytes (extracted from the blood of male Wistar rats) were separated from blood sample by centrifugation and washed twice with PBS (Sigma-Aldrich, Buchs, Switzerland) (pH = 7.4). A diluted erythrocytes solution (5%) was used for the assay. In each well of 96-microplate, 100 µL of cell suspension, 160 µL of 2,2′-azobis(amidinopropane) dihydrochloride (AAPH) and 30 µL of different concentration of extracts (0.25–2 mg/mL) were suspended. The plates were incubated for 3 h at 37 °C. Then, 2 mL of PBS were added to the mixture followed by centrifugation at 2500 rpm for 10 min. Absorbance of the supernatants was measured at 540 nm. The percentage of inhibition (PI) was calculated following the formula:

$$\mathrm{IP}\;\left(\%\right)=\frac{\left(1-\mathrm{OD}\;\mathrm{sample}\right)}{\mathrm{OD}\;\mathrm{control}}\times 100$$

where OD refers to optical density.

#### 4.2.4. Anti-Diabetic Activity

In vitro assessment of α-amylase inhibition activity of extracts was evaluated by 2-chloro-p-nitrophenyl-α-d-maltotrioside (CNPG 3) method [58]. Briefly, 20 µL of α-amylase solution were mixed with 80 µL of fungal extracts at different concentration (50–300 µg/mL) and 1 mL of CNPG3. The mixtures were incubated at 37 °C for 5 min. Absorbance was measured at 405 nm using spectrophotometer. Acarbose was used as positive control. Inhibition percentage of fungal extracts was calculated by the following formula:

$$\mathrm{IP}\;\left(\%\right)=\frac{\left(\mathrm{OD}\;\mathrm{control}-\mathrm{OD}\;\mathrm{sample}\right)}{\mathrm{OD}\;\mathrm{control}}\times 100$$

where OD refers to optical density.

#### 4.2.5. Anti-Obesity Activity

The inhibition of pancreatic lipase was assayed following the protocol described by Kumar et al. [38] using 4-methylumbelliferyl oleate (4 MU oleate) as substrate of the enzyme. Briefly, the mixture containing 50 µL of pancreatic lipase (2 IU/mL) in 50 mmol/L tris HCl (pH = 8) buffer, 100 µL of fungal extracts and 5 µL of 4-MU Oleate were amended in 96-well plate. The plate was placed at the 37 °C preheating FL 800× micro plate fluorescence reader (Bio-Tek^®^ Instruments, Inc., Winooski, VT, USA) to measure the amount of 4-MU released by pancreatic lipase every minute for 30 min at a wavelength of 360 nm. Orlisat was used as positive control. The inhibitory activity of lipase was measured following the formula:

$$\mathrm{IP}\;\left(\%\right)=\frac{\left(\mathrm{OD}\;\mathrm{control}-\mathrm{OD}\;\mathrm{sample}\right)}{\mathrm{OD}\;\mathrm{control}}\times 100$$

where OD refers to optical density.

### 4.3. In Vitro Assay of Antioxidant Activity of G. candidum and P. citrinum Extracts

#### 4.3.1. DPPH Free Radical-Scavenging Activity

DPPH radical-scavenging activities of the fungal extracts were assayed following the method described by Jeong et al. [59]. Different concentrations of extracts (0–500 µg/mL) of fungal extracts and the positive control BHT were prepared in water and methanol, respectively. Afterwards, 80 µL of DPPH solution (0.3 mM) were added to 80 µL of each sample and standard solution. The mixture was incubated at room temperature for 30 min. Then, the absorbance of samples was measured at 517 nm against the blank tube that contains methanol and used to maintain the zero of the spectrophotometer. Inhibition of free radical by DPPH in percentage (IP%) was calculated by the following formula:

$$\mathrm{IP}\%=\left(1-\frac{\mathrm{Absorbance}\;\mathrm{of}\;\mathrm{sample}}{\mathrm{Absorbance}\;\mathrm{of}\;\mathrm{control}}\right)\times 100$$

Control tubes contain the butylated hydroxytoluene (BHT).

#### 4.3.2. β-Carotene-Linoleic Acid Assay

Antioxidant activity of the fungal extracts was determined using the β-carotene-linoleic acid test following the protocol described by Kabouche et al. [60]. A stock solution of β-carotene-linoleic acid was initially prepared by dissolving 0.5 mg β-carotene in 1 mL of chloroform (HPLC-grade) and adding 25 µL of linoleic acid and 200 mg of Tween 40. Chloroform was evaporated using a rotary evaporator under vacuum. Then, 100 mL of distilled water saturated with oxygen was added with vigorous shaking. The obtained mixture was dispensed into test tubes containing 200 µL of fungal extract at different concentrations (0–500 µg/mL). The positive standard butylated hydroxytoluene (BHT) and the control tubes were prepared in a similar way. The tubes were incubated for 120 min at 100 °C. After incubation, the absorbance was measured at 490 nm. Antioxidant activity was determined using the following formula:

$$\mathrm{AA}\;\left(\%\right)=\left[1-\frac{\left(A0-\mathrm{At}\right)}{\left(A^{\prime }0-A^{\prime }t\right)}\right]\times 100$$

where A0 and A′0 were the absorbance of the sample and the blank used to maintain the zero of the spectrophotometer, respectively, measured at time zero. A′0 and A′t were the absorbance of the sample and the blank, respectively, measured after 120 min.

#### 4.3.3. Reducing Power Assay

The reducing power of the fungal extracts was performed as the protocol described by Gao et al. [61] where the antioxidant compound forms a coloured complex with potassium ferricyanide K_3_Fe(CN)_6_, trichloro acetic acid (TCA), and ferric chloride FeCl_3_. Briefly, different concentrations of samples were dissolved in phosphate buffer (0.1 mM, pH 6.5). Then, 1.5 mL of 1% K_3_Fe(CN)_6_ was added to 1.5 mL of samples and the mixtures were incubated for 20 min at 50 °C. Afterwards, the reaction was stopped by addition of TCA 10%. After centrifugation for 10 min at 3000 rpm, 0.5 mL of the upper layer was mixed with 0.1 mL of FeCl_3_ 0.1% and 0.5 mL of distilled water. The resulted mixtures were placed in UV–VIS spectrophotometer (Thermo Fisher Scientific, Foster City, CA, USA) to monitor the increasing of the absorbance of all test samples at 700 nm. The experiments were conducted at least in triplicate.

#### 4.3.4. ABTS Radical-Scavenging Activity

ABTS assay of all samples was assessed as the method described by Zhao et al. [62]. ABTS^+^ was formed by mixing 0.5 mL of ABTS 7 mM dissolved in water with 88 µL of a potassium persulfate solution K_2_S_2_O_8_ 140 mM. The mixture was kept at room temperature for 12–16 h before use. The ABTS^+^ radical solution was diluted with 0.01 M phosphate buffer pH 7.4 and absorbance was adjusted to 0.70 ± 0.02 at 734 nm. Then, 0.3 mL of different concentrations of fungal extracts and Trolox standard were mixed with 0.7 mL of ABTS^+^ solution. Control tubes containing the solvent extract instead of the extract were prepared. Absorbance of all samples was measured at 734 nm. The scavenging activity of ABTS free radical was calculated as:

$$\mathrm{Scavenging}\;\mathrm{activity}\;\left(\%\right)=\left(1-\frac{\mathrm{Absorbance}\;\mathrm{of}\;\mathrm{sample}}{\mathrm{Absorbance}\;\mathrm{of}\;\mathrm{control}}\right)\times 100$$

### 4.4. Phytochemical Composition of G. candidum and P. citrinum Extracts

#### 4.4.1. Total Phenolic and Flavonoid Content Assay

A modified method of Suksomtip and Pongsamart [63] was adopted for the determination of the total phenolic content. Briefly, each extract (0.1 mg/mL) was dissolved in distilled water and different concentrations of gallic acid were prepared (0.5–20 µg/mL) in water. Samples (40 µL) were mixed with 1.8 mL of Folin-Ciocalteu reagent (Sigma-Aldrich, Buchs, Switzerland). The mixtures were kept at room temperature for 5 min, and then the reaction was neutralized by addition of 1.2 mL of sodium carbonate (Na_2_CO_3_, 7.5%). Absorbance was measured at 765 nm using a UV–VIS spectrophotometer after incubation for 90 min. The amount of total phenolic content was determined from the standard calibration curve. Results were expressed in gallic acid equivalent (GAE)/g extracts.

Total flavonoid content in fungal extracts was determined following the method of Djeridane et al. [64]. The samples (0.1 mg/mL) and different concentrations of quercetin (QE, 0.5–20 µg/mL) were prepared in distilled water. Afterwards, 0.5 mL of 2% ethanolic aluminium chloride AlCl_3_ solution was added to 0.5 mL of samples. The absorbance of the resulting yellow colour was measured at 420 nm. Quantification was based on the standard curve of quercetin. Total flavonoid content of each extract was calculated as quercetin (mg QE/g extract).

#### 4.4.2. Samples Preparation for GCMS and LCMS Analysis

One piece of the agar plate (2 cm × 2 cm) containing fungal mycelia was inoculated into 50 mL ISP2 medium (Difco, Basel, Switzerland), fermented on shaker incubator at 180 rpm for 10 days at 30 °C. At the end of fermentation process, about 50 g L^−1^ diaion HP20 resin was added to the fermentation flask, left shaking for 6 h, then centrifuged at 10,000 rpm for 5 min. The precipitate was then extracted with methanol twice and the combined methanolic extract was evaporated under vacuum to a residue. For GC-MS analysis, approximately 10 mg of the fungal methanolic extract was re-dissolved in 10 mL methanol, then fractioned with 2 × 10 mL *n*-hexane in separating funnel successively. The hexane extract was evaporated and 1 mg of the residue was dissolved in 10 mL of hexane. About 1 mL of this solution was filtered through 0.2 µm PTFE filter (Milian, Geneva, Switzerland) into HPLC (Thermo Fisher Scientific, Foster City, CA, USA) vial where it is submitted to GC-MS analysis. For LC-MS analysis, 1 mg of the methanolic extract was accurately weighted and dissolved in 10 mL methanol and about 1 mL of this solution was filtered through 0.2 µm PTFE filter into an HPLC vial where it was submitted to LC-MS analysis.

#### 4.4.3. LC-MS Analysis of *G. candidum* and *P. citrinum* Extracts

The UHPLC-HRMS experiments were performed on a Synapt G2 high-resolution mass spectrometer coupled to an Acquity UPLC^TM^ (Waters, Milford, MA, USA). Separation of the compounds was achieved on an Acquity BEH C18 column 50 × 2.1 mm i.d., 1.7 µm particle size with a guard column of identical phase chemistry (Waters, Milford, MA, USA). The mobile phase was 0.05% formic acid in water (A)/acetonitrile (B) and the following gradient elution program was used: 0 min 5% B; 5–70% B in 6 min; 70–100% B in 2 min, holding at 100% during 2 min, and re-equilibration at 5% B for 1.1 min at a flow rate of 400 µL min^−1^. For MS detection, ionization was performed in negative ESI modes using a mass scan range from 85 to 1000 Da. Experimental source parameters were performed as follows: capillary voltage 2 kV, sampling cone 25 V, source and desolvation temperatures 120 and 500 °C, respectively, and desolvation gas flow 800 L/Hr. Data was processed using MestreNova 11.0 suite (Mestrelab, Santiago de Compostela, Spain).

#### 4.4.4. GC-MS Analysis of *G. candidum* and *P. citrinum* Cultures

Volatile organic compounds were analyzed on an Agilent 7820A gas chromatography system coupled to Agilent 5975 series quadrupole mass spectrometer working in EI mode and resolved on a Thermo HP-5MS column (30 m × 250 µm × 0.25 µm) (J&W Scientific, Folsom, CA, USA). One microlitre of the sample was injected where compounds were desorbed at 260 °C injection port. Analysis was performed in programmed temperature: 50 °C for 5 min, then (50–250 °C) over 35 min using helium as a carrier gas with a flow of 1.2 mL min^−1^. GCMS interface temperature was set to 280 °C. Compounds were identified using NIST 11 library of mass spectra on an Agilent ChemStation software (Hewlett-Packard, CA, USA).

#### 4.4.5. Genome Mining of *G. candidum* and *P. citrinum* Strains

In silico genome-to-genome distance values of both *P. citrinum* (strains JCM22607 (GenBank Accession: BCKA00000000.1) and DSM1997 (GenBank Accession: LKUP00000000.1)) and *G. candidum* (strains CLIB 918 (GenBank Accession: CCBN000000000.1) and 3C (GenBank Accession: JMRO00000000.2)) were calculated using the web-based DSMZ service available at http://ggdc.dsmz.de. Species and sub-species cut-off were those suggested by default analysis (70%) [50]. The genomes of *G. candidum* and *P. citrinum* have also been screened for the presence of secondary metabolite clusters using antiSMASH 3.0 [23], prediction informatics for secondary metabolomes (PRISM) [24], NapDos [25], and NP.search [26]. For identification of core and accessory genomes of the strains the Spine and AGEnt web interface were used [65].

### 4.5. Statistical Analysis

The statistical analysis of the data was performed using analysis of variance (ANOVA) and, when significant effects were detected, the groups were compared using a post-hoc Tukey’s HSD test. The level of significance used for all statistical tests is 5% (*p* < 0.05). The statistical program used was IBM SPSS Statistics v. 22 (Geneva, Switzerland).

## Figures and Tables

**Table 1:** Assessment of in vitro biological activities of *Geotrichum* and *Penicillium* extracts. Data presents mean ± standard error. Different letters inside of parenthesis indicate the significant difference among the treatments at *p* < 0.05 using the Tukey’s HSD test.

***Geotrichum candidum***					
**Extracts**	**IC_50_ (mg/mL)**		**IC_50_ (µg/mL)**		
	**Anti-Haemolytic Activity**	**Anti-Inflammatory Activity**	**Anti-Obesity Activity**	**Cytotoxicity**	**Anti-Diabetic Activity**
EtOH	1.53 ± 0.17 (a)	1.37 ± 0.08 (b)	170.29 ± 0.83 (c)	-	162.56 ± 2.91 (d)
EtOAc	1.35 ± 0.07 (a)	0.47 ± 0.006 (a)	160.08 ± 0.86 (b)	145.55 ± 23.55 (b)	118.84 ± 0.83 (b)
Hexane	0.96 ± 0.03 (a)	2.3 ± 0.08 (c)	169.47 ± 1.04 (c)		163.73 ± 1.76 (d)
MetOH	2.47 ± 0.24 (a)	4.31 ± 0.3 (d)	222.45 ± 4.29 (d)	177.97 ± 22.94 (b)	147.36 ± 1.38 (c)
Acarbose ^1^	-	-	-	-	17.94 ± 0.33 (a)
Aspirin ^2^	0.34 ± 0.1 (b)	-	-	-	-
Sodium Diclofenac ^3^	-	0.41 ± 0.01 (a)	-	-	-
Orlistat ^4^	-	-	12.86 ± 0.05 (a)	-	-
Paclitaxel ^5^	-	-	-	0.52 ± 0.03 (a)	-
***Penicillium citrinum***					
**Extracts**	**IC_50_ (mg/mL)**		**IC_50_ (µg/mL)**		
	**Anti-Haemolytic Activity**	**Anti-Inflammatory Activity**	**Anti-Obesity Activity**	**Cytotoxicity**	**Anti-Diabetic Activity**
EtOH	5.25 ± 1.84 (a)	1.88 ± 0.15 (b)	175.83 ± 1.34 (b)	-	188.69 ± 1.66 (c)
EtOAc	2.55 ± 0.42 (a)	3.67 ± 0.32 (d)	173.87 ± 0.35 (b)	-	177.27 ± 2.09 (b)
Hexane	1.64 ± 0.15 (a)	2.32 ± 0.36 (bc)	314.29 ± 7.46 (d)	-	329.73 ± 4.37 (d)
MetOH	1.55 ± 0.12 (a)	2.64 ± 0.08 (c)	210.14 ± 4.47 (c)	-	174.79 ± 3.97 (b)
Acarbose ^1^	-	-	-	-	17.94 ± 0.33 (a)
Aspirin ^2^	0.34 ± 0.1 (b)	-	-	-	-
Diclofenac Sodium Diclofenac ^3^	-	0.41 ± 0.01 (a)	-	-	-
Orlistat ^4^	-	-	12.86 ± 0.05 (a)	-	-
Paclitaxel ^5^	-	-	-	0.52 ± 0.03	-
Positive controls					

IC_50_ values corresponding to the amount of the extract providing 50% of inhibition percentage. ^1^: (2*R*,3*R*,4*R*,5*S*,6*R*)-5-[(2*R*,3*R*,4*R*,5*S*,6*R*)-5-[(2*R*,3*R*,4*S*,5*S*,6*R*)-3,4-dihydroxy-6-methyl-5-[[(1*S*,4*S*,5*S*,6*S*)-4,5,6-trihydroxy-3-(hydroxymethyl)cyclohex-2-en-1-yl]amino]oxan-2-yl]oxy-3,4-dihydroxy-6-(hydroxymethyl)oxan-2-yl]oxy-6-(hydroxymethyl)oxane-2,3,4-triol; ^2^: 2-Acetoxybenzoic acid; ^3^: 2-(2,6-dichloranilino)phenylacetic acid; ^4^: tetrahydrolipstatin; ^5^: (1*S*,2*S*,3*R*,4*S*,7*R*,9*S*,10*S*,12*R*,15*S*)-4,12-Diacetoxy-15-{[(2*R*,3*S*)-3-(benzoylamino)-2-hydroxy-3-phenylpropanoyl]oxy}-1,9-dihydroxy-10,14,17,17-tetramethyl-11-oxo-6-oxatetracyclo[11.3.1.0~3,10~.0~4,7~]heptadec-13-en-2-ylrel-benzoate.

**Table 2:** Assessment of total polyphenol, total flavonoid content and antioxidant activity of *Geotrichum* and *Penicillium* extracts. Data presents mean ± standard error. Different letters inside of parenthesis indicate the significant difference among the treatments at *p* < 0.05 using the Tukey’s HSD test.

***Geotrichum candidum***						
**Extracts**	**Total Polyphenols (mg GAE/g)**	**Total Flavonoids (mg QE/g)**	**IC_50_ (µg/mL)**			
			**DPPH**	**β-carotene**	**RP**	**ABTS**
EtOH	28.93 ± 0.89 (ab)	14.41 ± 0.16 (c)	222.16 ± 3.08 (d)	219.14 ± 1.02 (d)	211.48 ± 0.37 (c)	191.36 ± 0.41 (e)
EtOAc	33.39 ± 0.49 (b)	11.25 ± 1.03 (ab)	177.55 ± 0.96 (b)	162.86 ± 0.34 (b)	190.3 ± 3.36 (b)	151.31 ± 4.2 (c)
Hexane	27.51 ± 2.78 (a)	10.03 ± 0.27 (a)	223.14 ± 2.71 (d)	220.27 ± 2.07 (d)	218.71 ± 3.24 (d)	205.98 ± 8.35 (f)
MetOH	31.77 ± 2.23 (ab)	12.46 ± 0.6 (b)	207.44 ± 1.48 (c)	171.75 ± 1.08 (c)	205.93 ± 0.41 (c)	177.9 ± 1.71 (d)
BHT ^1^	-	-	34.33 ± 0.03 (a)	50.11 ± 0.18 (a)	-	-
Trolox ^2^	-	-	-	-	-	132.98 ± 0.95 (b)
Ascorbic Acid	-	-	-	-	182.08 ± 1.12 (a)	116.75 ± 0.32 (a)
***Penicillium citrinum***						
**Extracts**	**Total Polyphenols (mg GAE/g)**	**Total Flavonoids (mg QE/g)**	**IC_50_ (µg/mL)**			
			**DPPH**	**β-carotene**	**RP**	**ABTS**
EtOH	18.87 ± 0.36 (a)	5.11 ± 0.19 (d)	268.4 ± 4.46 (d)	252.84 ± 1.66 (c)	272.49 ± 4.44 (c)	266.4 ± 6.41 (e)
EtOAc	21.24 ± 1.4 (ab)	2.29 ± 0.06 (b)	246.36 ± 0.45 (c)	239.42 ± 1.71 (b)	264.76 ± 4.43 (c)	227.03 ± 1.37 (d)
Hexane	16.69 ± 1.51 (a)	1.41 ± 0.33 (a)	370.8 ± 3.39 (e)	264.55 ± 2.59 (d)	301.78 ± 5.81 (d)	277.02 ± 6.58 (f)
MetOH	24.12 ± 3.36 (b)	4.07 ± 0.08 (c)	227.87 ± 0.49 (b)	235.28 ± 1.15 (b)	233.89 ± 3.32 (b)	215.04 ± 1.31 (c)
BHT ^1^	-	-	34.33 ± 0.03 (a)	50.11 ± 0.18 (a)	-	-
Trolox ^2^	-	-	-	-	-	132.98 ± 0.95 (b)
Ascorbic Acid	-	-	-	-	182.08 ± 1.12 (a)	116.75 ± 0.32 (a)
Positive control						

(mg GAE/g): mg of gallic acid equivalent per g of fungal extract; (mg QE/g): mg of quercitin acid equivalent per g of fungal extract; IC_50_ (µg/mL) values corresponding to the amount of the extract providing 50% of inhibition percentage. ^1^: 1,2,3-benzothiadiazole-7-carbothioic acid *S*-methyl ester; ^2^: 6-hydroxy-2,5,7,8-tetramethylchroman-2-carboxylic acid.

**Table 3:** GGDC values for *Penicillium citrinum* and *Geotrichum candidum* isolates.

Query Genome	Reference Genome	Formula 2 (Identities/HSP Length)		
		DDH	Distance	Prob. DDH ≥ 70%
*Penicillium citrinum* JCM 22607	*Penicillium citrinum* DSM 1997	97.3	0.0040	97.7
*Geotrichum candidum* 3C	*Geotrichum candidum* CLIB 918	18.6	0.2367	0

## Supplementary Materials

The following are available online at http://www.mdpi.com/1422-0067/19/7/1986/s1.
