# Supplementary material for: Date Palm Trees Root-Derived Endophytes as Fungal Cell Factories for Diverse Bioactive Metabolites
Source: Int J Mol Sci. 2018 Jul 7;19(7):1986. doi: 10.3390/ijms19071986 (PMC6073733; doi:10.3390/ijms19071986)
Supplement: Supplementary file 1 [file ijms-19-01986-s001.pdf]

Supplementary Table 1. LC-MS analysis of *Geotrichum candidum* extracts.

Rt	HRESIMS	Tentative identification	Formula of the molecule	Structure of the molecule	References					
					Antimicrobial Activity	Anti-diabetic activity	Anti-hemolysis activity	Anti-inflammatory activity	Anti-obesity activity	Cytotoxicity
5.60	271.1440	Clavicipitic acid	$C_{16}H_{18}N_2O_2$	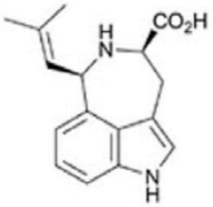	[1]	-	-	-	-	[2]
6.24	305.1745	7-butyl-6,8-dihydroxy-3-pent-11-enylisochrom en-1-one	$C_{18}H_{24}O_4$	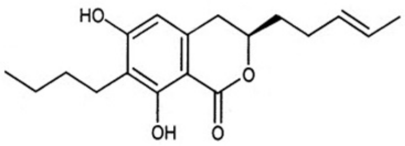	[3]	-	-	-	-	[4]
6.61	303.1591									
6.70	211.1444	Cyclo(L-Leu-L-Pro)	$C_{11}H_{18}N_2O_2$	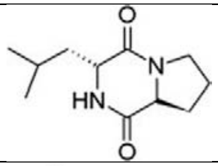	[5]	-	-	[6]	-	[7]
6.80	261.1235	Cyclo(L-Tyr-L-Pro)	$C_{14}H_{16}N_2O_3$	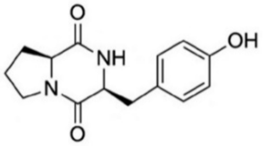	[8]	-	-	[9]	-	[10]
6.92	197.1286	Cyclo-(L-Pro-L-Val)	$C_{10}H_{16}N_2O_2$	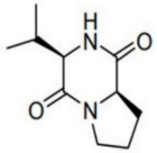	[5]	-	-	[11]	-	[12]

Supplementary Table 1. Continued.

Rt	HRESIMS	Tentative identification	Formula of the molecule	Structure of the molecule	References					
					Antimicrobial Activity	Anti-diabetic activity	Anti-hemolysis activity	Anti-inflammatory activity	Anti-obesity activity	Cytotoxicity
7.00	317.1384	8-Methoxytrypethelone methyl ether	$C_{18}H_{20}O_5$	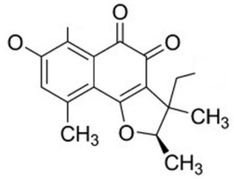	[13]	-	-	-	-	[13]
7.10	351.1228	7-methoxy-4,8,9-trihydroxy-1,6,7,8-tetrahydro-2H-benzo[j]fluoranthen-3-one	$C_{21}H_{18}O_5$	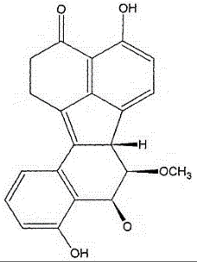	-	-	-	-	-	[14]
7.20	411.1552	Brasilamide F	$C_{22}H_{22}N_2O_6$	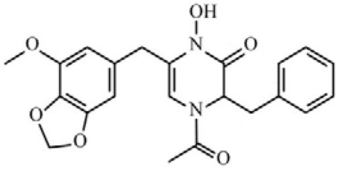	[15]	-	-	-	-	[16]
7.27	273.1455	Cordiachrome D	$C_{17}H_{20}O_3$	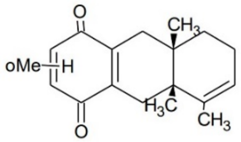	[17]	-	-	-	-	[18]
7.37	329.2324	Sch-725674	$C_{18}H_{32}O_5$	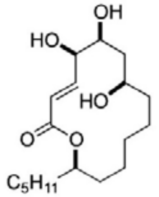	[19]	-	-	-	-	-

Supplementary Table 1. Continued.

Rt	HRESIMS	Tentative identification	Formula of the molecule	Structure of the molecule	References					
					Antimicrobial Activity	Anti-diabetic activity	Anti-hemolysis activity	Anti-inflammatory activity	Anti-obesity activity	Cytotoxicity
7.47	312.1232	Carbonarin E	$C_{18}H_{17}NO_4$	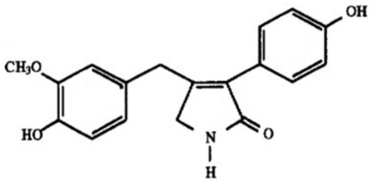	[20]	-	-	-	-	[20]
7.59	463.2693	Virescenoside E	$C_{26}H_{38}O_7$	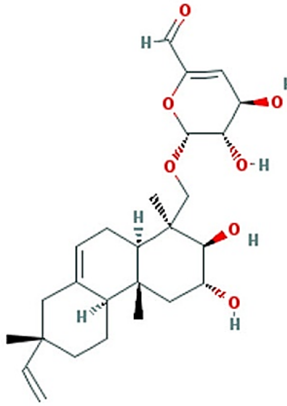	-	-	[21]	-	-	[21]
7.72	452.2792	Cytochalasin J	$C_{28}H_{37}NO_4$	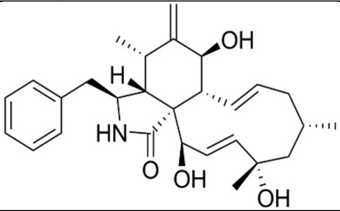	[22]	[23]	[24]	[24]	-	[25, 26]
7.41	257.1494	2-Methylenecycloheptene-1,3-diglycine	$C_{12}H_{20}N_2O_4$	-	-	-	-	-	-	-

Supplementary Table 1. Continued.

Rt	HRESIMS	Tentative identification	Formula of the molecule	Structure of the molecule	References					
					Antimicrobial Activity	Anti-diabetic activity	Anti-hemolysis activity	Anti-inflammatory activity	Anti-obesity activity	Cytotoxicity
7.79	423.2745	β-Hydroxy Mevinolin	$C_{24}H_{38}O_6$	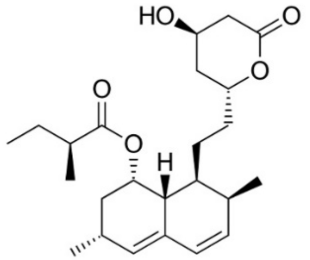	[27]	-	-	-	[28]	[29]
7.88	421.2587	g-Hydroxy Mevinolin								
7.97	325.1547	Cyclo(Phenylalanyl-N-methyltyrosyl)	$C_{19}H_{20}N_2O_3$	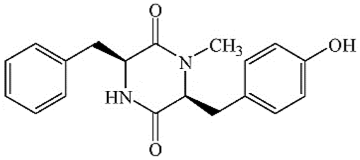	[30]	-	-	-	-	-
8.09	412.3575	14-Aza-24-methylene-D-homocholesta-8,14-dien-3-ol	$C_{28}H_{45}NO$	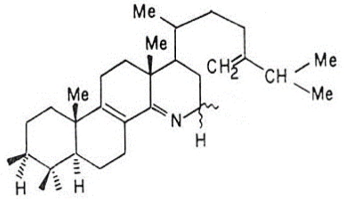	[31]	-	-	-	-	-
8.18	440.3885	4,4-Dimethyl-3-hydroxy-24-methylene-14a-aza-D-homo- 5a-cholesta-8,14-diene	$C_{30}H_{49}NO$	-	-	-	-	-	-	-
8.30	454.3678	Acetoxy-24-methylene-14a-aza-n-homo-5a-cholesta-8,14-diene	$C_{30}H_{47}NO_2$	-	[32]	-	-	-	-	-

Supplementary Table 1. Continued.

Rt	HRESIMS	Tentative identification	Formula of the molecule	Structure of the molecule	References					
					Antimicrobial Activity	Anti-diabetic activity	Anti-hemolysis activity	Anti-inflammatory activity	Anti-obesity activity	Cytotoxicity
8.63	410.3416	3-Keto-24-methylene-14a-aza-D-homo-5a-cholesta-8, 14-diene	$C_{28}H_{43}NO$	-	[33]	-	-	-	-	-
8.89	426.3732	4a-Methyl-15-aza-24-methylene-D-homocholesta-8,14-dien-3-ol	$C_{29}H_{47}NO$	-	[34]	-	-	-	-	-

Supplementary Table 2. LC-MS analysis of *Penicillium citrinum* extracts.

Rt	HRESIMS	Tentative identification	Formula of the molecule	Structure of the molecule	References					
					Antimicrobial Activity	Anti-diabetic activity	Anti-hemolysis activity	Anti-inflammatory activity	Anti-obesity activity	Cytotoxicity
7.80	158.1177	1,2-Dihydroxyindolizidine	$C_8H_{15}NO_2$	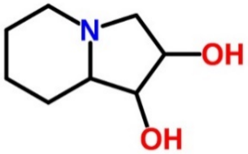	[35]	-	-	-	-	-
9.11	137.1075	2-Isopropyl-6-methylpyrazine	$C_8H_{12}N_2$	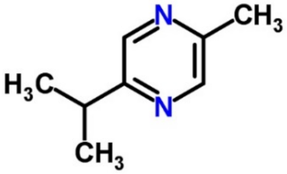	-	-	-	[36]	-	-
9.37	197.1285	Cyclo-(L-Pro-L-Val)	$C_{10}H_{16}N_2O_2$	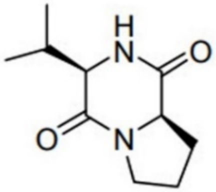	[5]	-	-	[11]	-	[12]
10.75	185.1286	Cyclo(L-Ala-L-Leu)	$C_9H_{16}N_2O_2$	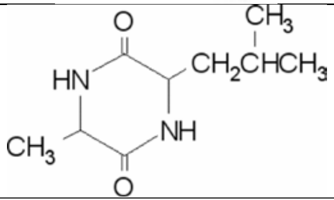	-	-	-	-	-	-
12.11	261.1235	Cyclo(L-Tyr-L-Pro)	$C_{14}H_{16}N_2O_3$	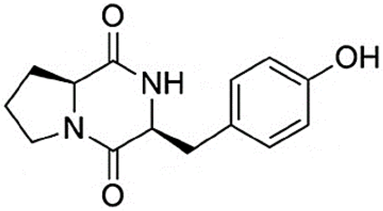	[8]	-	-	[9]	-	[10]

Supplementary Table 2. Continued.

Rt	HRESIMS	Tentative identification	Formula of the molecule	Structure of the molecule	References					
					Antimicrobial Activity	Anti-diabetic activity	Anti-hemolysis activity	Anti-inflammatory activity	Anti-obesity activity	Cytotoxicity
12.64	211.1440	Cyclo(L-Leu-L-Pro)	$C_{11}H_{18}N_2O_2$	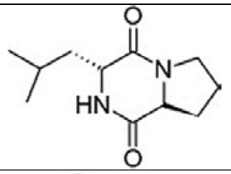	[37]	-	-	[6]	-	[7]
13.64	245.1283	Cyclo(L-Phe-L-Pro)	$C_{14}H_{16}N_2O_2$	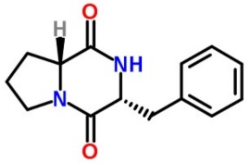	[38]	-	-	-	-	-
14.88	243.0875	Lumichrome	$C_{12}H_{10}N_4O_2$	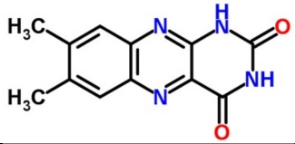	[39]	-	-	-	-	-
15.04	247.1438	Cyclo(L-Val-L-Phe)	$C_{14}H_{18}N_2O_2$	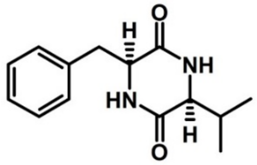	[40]	-	-	-	-	-
15.74	227.1754	L-Leucine anhydride	$C_{12}H_{22}N_2O_2$	-	-	-	-	-	-	-
17.43	309.0868	Chaetominedione	$C_{17}H_{12}N_2O_4$	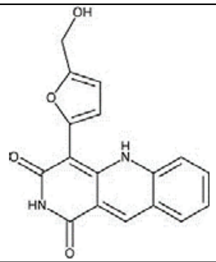	[41]	-	-	-	-	[42]

Supplementary Table 2. Continued.

Rt	HRESIMS	Tentative identification	Formula of the molecule	Structure of the molecule	References					
					Antimicrobial Activity	Anti-diabetic activity	Anti-hemolysis activity	Anti-inflammatory activity	Anti-obesity activity	Cytotoxicity
18.73	391.2840	Merulinic acid B	$C_{24}H_{38}O_4$	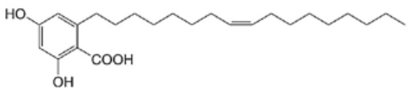	[43]	-	-	-	-	-
19.65	430.1608	Amicycline	$C_{21}H_{23}N_3O_7$	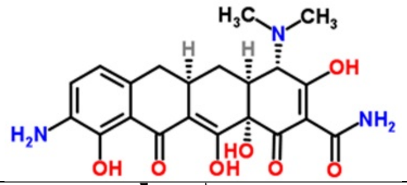	[44]	-	-	-	-	-
21.92	251.0912	Citrinin	$C_{13}H_{14}O_5$	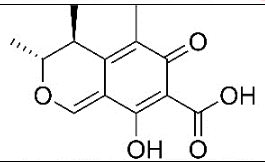	[45]	-	-	[46]	-	[45]
23.06	267.1225	5-Hydroxyvertinolide	$C_{14}H_{18}O_5$	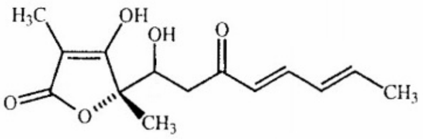	[47]	-	-	[48]	-	-

Supplementary Table 3. GC-MS analysis results of *Penicillium citrinum*.

Rt	Tentative identification	Kovat's Index	Lib Score	Formula of the molecule	Structure of the molecule	References					
						Antimicrobial Activity	Anti-diabetic activity	Anti-hemolysis activity	Anti-inflammatory activity	Anti-obesity activity	Cytotoxicity
3.432	α-pinene	940	90.5	$C_{10}H_{16}$	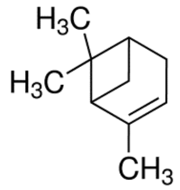	[49]	-	-	[50]	[51]	-
4.576	Camphene	942	86.9	$C_{10}H_{16}$	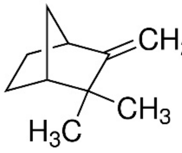	[52]	-	-	[53]	[54]	[52]
4.748	Trans-4,5-epoxy-carane	948	95.1	$C_{10}H_{16}O$	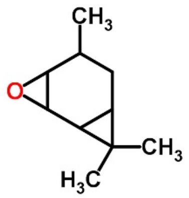	-	-	-	-	-	-
5.515	Isopropyliden e cyclohexane	955	85.9	C_9H_{16}	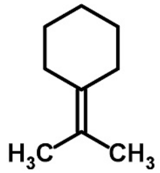	-	-	-	-	-	-
6.102	3-Isopropenyl-5-methyl-1-cyclohexene	990	92.1	$C_{10}H_{16}$	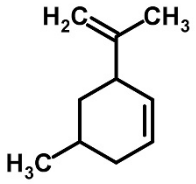	-	-	-	-	-	-

Supplementary Table 3. Continued.

Rt	Tentative identification	Kovat's Index	Lib Score	Formula of the molecule	Structure of the molecule	References					
						Antimicrobial Activity	Anti-diabetic activity	Anti-hemolysis activity	Anti-inflammatory activity	Anti-obesity activity	Cytotoxicity
6.184	Isocineole	1001	87.9	$C_{10}H_{18}O$	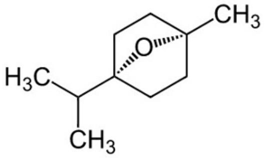	-	-	-	-	-	-
6.373	3-isobutyl cyclohexene	1013	89.5	$C_{10}H_{18}$	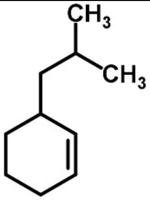	-	-	-	-	-	-
6.752	Phenylethanol	1082	91.0	$C_8H_{10}O$	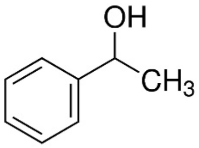	[55]	-	-	-	-	-
6.879	α-isophorone	1097	92.5	$C_9H_{14}O$	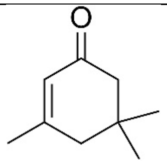	-	-	-	-	-	-
7.123	Acetylvaleric acid methyl ester	1120	93.2	$C_8H_{14}O_3$	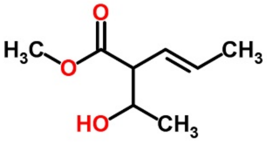	-	-	-	-	-	-

Supplementary Table 3. Continued.

Rt	Tentative identification	Kovat's Index	Lib Score	Formula of the molecule	Structure of the molecule	References					
						Antimicrobial Activity	Anti-diabetic activity	Anti-hemolysis activity	Anti-inflammatory activity	Anti-obesity activity	Cytotoxicity
7.682	Methyl 6-oxoheptanoate	1124	89.2	$C_8H_{14}O_3$	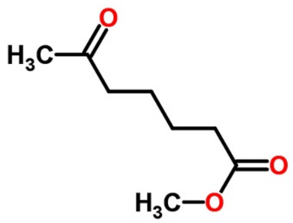	-	-	-	-	-	-
8.296	Citronellal	1132	87.2	$C_{10}H_{18}O$	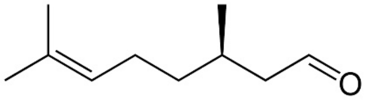	[56]	-	-	-	-	-
8.368	Isopulegol	1135	88.3	$C_{10}H_{18}O$	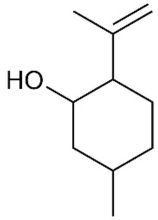	-	-	-	-	-	-
9.587	cis-p-mentha-2,8-diene-1-ol	1140	88.5	$C_{10}H_{16}O$	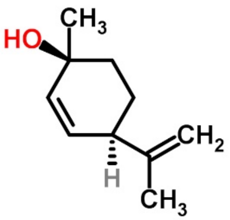	-	-	-	-	-	-

Supplementary Table 3. Continued.

Rt	Tentative identification	Kovat's Index	Lib Score	Formula of the molecule	Structure of the molecule	References					
						Antimicrobial Activity	Anti-diabetic activity	Anti-hemolysis activity	Anti-inflammatory activity	Anti-obesity activity	Cytotoxicity
10.006	1,2,3,4-tetrahydronaphthalene	1142	91.2	$C_{10}H_{12}$	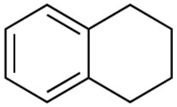	-	-	-	-	-	-
10.581	(3Z)-3-pentenyl benzene	1148	86.9	$C_{11}H_{14}$	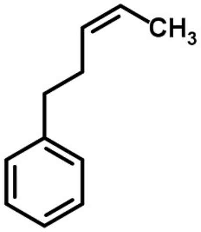	-	-	-	-	-	-
10.770	1-(2,2-dimethylcyclohexyl) ethanone	1151	93.0	$C_{10}H_{18}O$	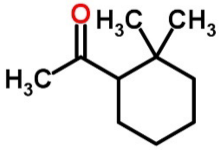	-	-	-	-	-	-
10.942	Norleucine	1153	90.0	$C_6H_{13}NO_2$	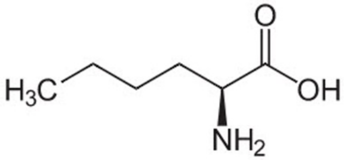	-	-	-	-	-	-
12.765	Arginine	1157	92.1	$C_6H_{14}N_4O_2$	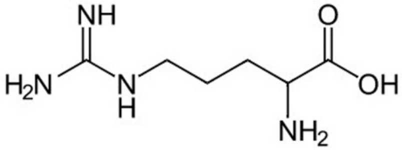	-	-	[57]	[58]	-	-

Supplementary Table 3. Continued.

Rt	Tentative identification	Kovat's Index	Lib Score	Formula of the molecule	Structure of the molecule	References					
						Antimicrobial Activity	Anti-diabetic activity	Anti-hemolysis activity	Anti-inflammatory activity	Anti-obesity activity	Cytotoxicity
12.901	4-isopropyl-1-methyl-7-azabicyclo[4.1.0]heptane	1159	91.6	$C_{10}H_{19}N$	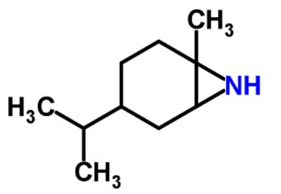	-	-	-	-	-	-
13.027	Terpinen-4-ol	1161	91.5	$C_{10}H_{18}O$	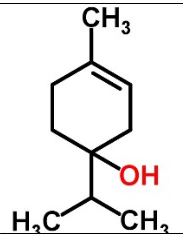	[59]	-	-	[60]	-	-
13.163	1,2,3,6-tetramethylbicyclo[2.2.2]octa-2,5-diene	1168	90.4	$C_{12}H_{18}$	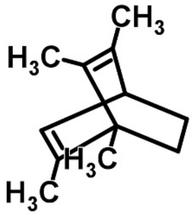	-	-	-	-	-	-
13.578	Dihydrocarvone	1179	89.5	$C_{10}H_{16}O$	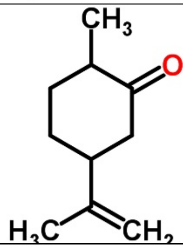	-	-	-	-	-	-

Supplementary Table 3. Continued.

Rt	Tentative identification	Kovat's Index	Lib Score	Formula of the molecule	Structure of the molecule	References					
						Antimicrobial Activity	Anti-diabetic activity	Anti-hemolysis activity	Anti-inflammatory activity	Anti-obesity activity	Cytotoxicity
14.725	(2E,3Z)-2-ethylidene-6-methyl-3,5-heptadienal	1182	93.1	$C_{10}H_{14}O$	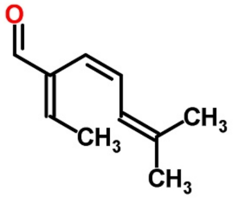	-	-	-	-	-	-
15.293	N-trimethylsilylaniline	1186	88.1	$C_9H_{15}NSi$	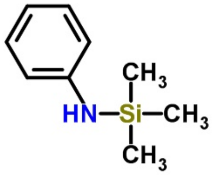	-	-	-	-	-	-
16.955	7-nonenoic acid methyl ester	1191	88.9	$C_{10}H_{18}O_2$	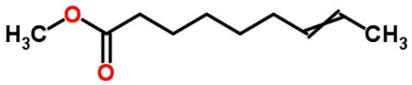	-	-	-	-	-	-
17.108	3-isopropenyl-2-methylcyclohexanol	1197	86.6	$C_{10}H_{18}O$	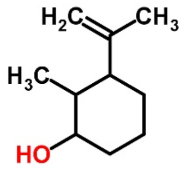	-	-	-	-	-	-
17.957	Trans-p-mentha-1(7),8-dien-2-ol	1201	91.5	$C_{10}H_{16}O$	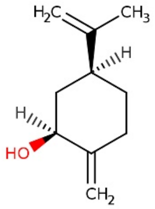	-	-	-	-	-	-

Supplementary Table 3. Continued.

Rt	Tentative identification	Kovat's Index	Lib Score	Formula of the molecule	Structure of the molecule	References					
						Antimicrobial Activity	Anti-diabetic activity	Anti-hemolysis activity	Anti-inflammatory activity	Anti-obesity activity	Cytotoxicity
21.586	1-methylverbenol	1209	85.9	$C_{12}H_{20}O$	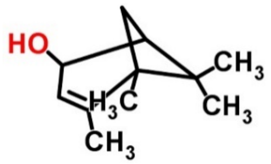	-	-	-	-	-	-
22.372	Cuminaldehyde	1214	91.9	$C_{10}H_{12}O$	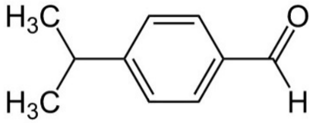	[61]	-	-	[61]	-	[61]
22.480	Citronellol epoxide	1220	91.5	$C_{10}H_{20}O$	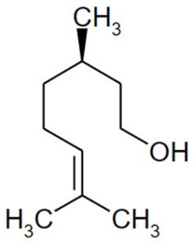	-	-	-	-	-	-
24.602	(1E)-1-ethylidene-7a-methyloctahydro-1H-indene	1239	93.1	$C_{12}H_{20}$	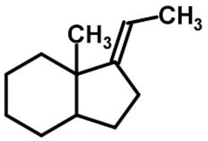	-	-	-	-	-	-
25.108	Isocarveol	1261	91.4	$C_{10}H_{16}O$	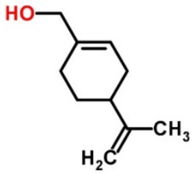	-	-	-	-	-	-

Supplementary Table 3. Continued.

Rt	Tentative identification	Kovat's Index	Lib Score	Formula of the molecule	Structure of the molecule	References					
						Antimicrobial Activity	Anti-diabetic activity	Anti-hemolysis activity	Anti-inflammatory activity	Anti-obesity activity	Cytotoxicity
25.451	3-methylindanone	1279	89.0	$C_{10}H_{10}O$	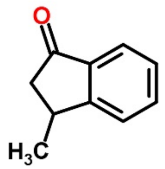	-	-	-	-	-	-
25.803	n-undecanal	1286	90.3	$C_{11}H_{22}O$	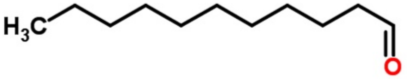	-	-	-	-	-	-
26.038	p-dimethylaminoacetophenone	1291	90.5	$C_{10}H_{13}NO$	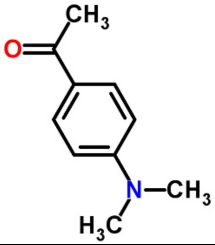	-	-	-	-	-	-
27.356	2,2,6,6-tetramethyl-4-piperidone	1306	92.1	$C_9H_{17}NO$	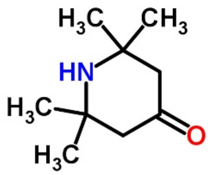	-	-	-	-	-	-
28.071	E-megastigma-4,6,8-triene	1317	87.9	$C_{13}H_{20}$	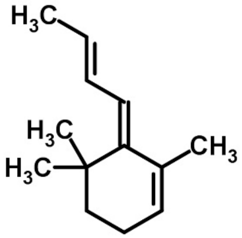	-	-	-	-	-	-

Supplementary Table 3. Continued.

Rt	Tentative identification	Kovat's Index	Lib Score	Formula of the molecule	Structure of the molecule	References					
						Antimicrobial Activity	Anti-diabetic activity	Anti-hemolysis activity	Anti-inflammatory activity	Anti-obesity activity	Cytotoxicity
31.220	4,8-dimethyl-3,7-nonadien-2-ol	1329	86.5	$C_{11}H_{20}O$	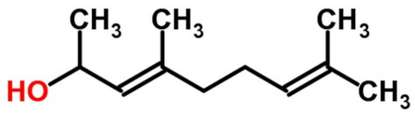	-	-	-	-	-	-
31.807	Decahydro-4a-methyl-1-naphthalenol	1363	90.5	$C_{15}H_{26}O$	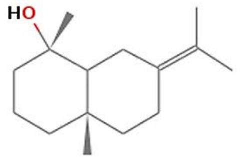	-	-	-	-	-	-
32.836	9-oxabicyclo[3.3.1]nonane-2,6-diol	1347	86.9	$C_8H_{14}O_3$	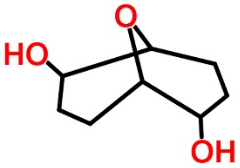	-	-	-	-	-	-
33.721	Octahydro-1-methyl-4(1H)-quinolinone	1386	91.5	$C_{10}H_{17}NO$	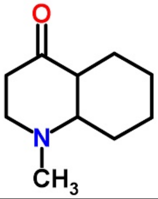	-	-	-	-	-	-

Supplementary Table 4. GC-MS analysis results of *Geotrichum candidum*.

Rt	Tentative identification	Kovat's Index	Lib Score	Formula of the molecule	Structure of the molecule	References					
						Antimicrobial Activity	Anti-diabetic activity	Anti-hemolysis activity	Anti-inflammatory activity	Anti-obesity activity	Cytotoxicity
6.175	1,4-dimethylpiperidine	820	87.6	$C_7H_{15}N$	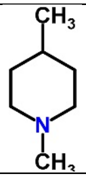	-	-	-	-	-	-
6.743	Isocineole	1001	89.2	$C_{10}H_{18}O$	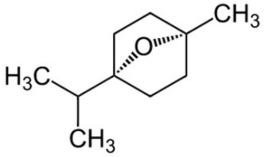	-	-	-	-	-	-
7.141	p-cymene	1011	90.6	$C_{10}H_{14}$	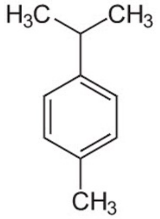	-	-	-	-	-	-
16.675	cis-4-octenoic acid methyl ester	1092	91.5	$C_9H_{16}O_2$	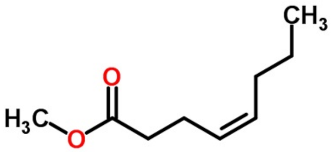	-	-	-	-	-	-
21.568	Trans-pulegone oxide	1230	90.2	$C_{10}H_{16}O_2$	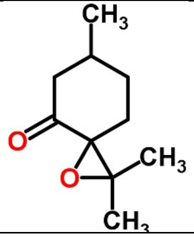	-	-	-	-	-	-

Supplementary Table 4. Continued.

Rt	Tentative identification	Kovat's Index	Lib Score	Formula of the molecule	Structure of the molecule	References					
						Antimicrobial Activity	Anti-diabetic activity	Anti-hemolysis activity	Anti-inflammatory activity	Anti-obesity activity	Cytotoxicity
22.498	Piperitone oxide	1237	94.1	$C_{10}H_{16}O_2$	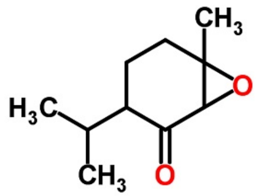	-	-	-	-	-	-
23.464	Ascaridole	1290	90.6	$C_{10}H_{16}O_2$	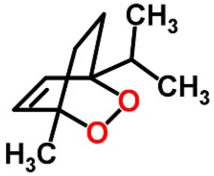	-	-	-	-	-	[62]
25.449	2(E)-undecenal	1341	91.9	$C_{11}H_{20}O$	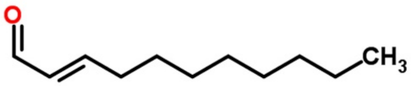	-	-	-	-	-	-
25.799	8-methylocta hydrocoumar in	1388	87.4	$C_{10}H_{16}O_2$	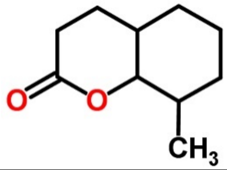	-	-	-	-	-	-
27.419	Iridomyrmeci n	1400	88.5	$C_{10}H_{16}O_2$	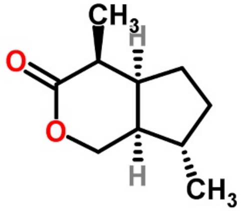	-	-	-	-	-	-

Supplementary Table 4. Continued.

Rt	Tentative identification	Kovat's Index	Lib Score	Formula of the molecule	Structure of the molecule	References					
						Antimicrobial Activity	Anti-diabetic activity	Anti-hemolysis activity	Anti-inflammatory activity	Anti-obesity activity	Cytotoxicity
28.954	1-isopropenyl-2,3,4,5-tetramethyl benzene	1412	90.1	$C_{13}H_{18}$	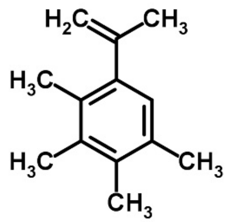	-	-	-	-	-	-
29.071	2,3-dihydro-3-methyl-3-benzofuran methanol	1414	85.1	$C_{10}H_{12}O_2$	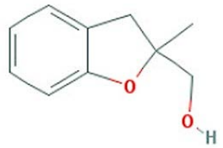	-	-	-	-	-	-
29.180	2-methyl-1,2,3,4-tetrahydro-1-naphthalenol	1418	88.1	$C_{11}H_{14}O$	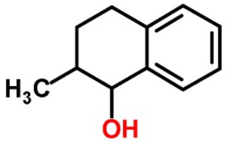	-	-	-	-	-	-
29.550	Capramide	1425	86.8	$C_{10}H_{21}NO$	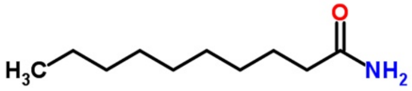	-	-	-	-	-	-
31.067	3-(hydroxymethyl)-5-methoxyphenol	1446	88.9	$C_8H_{10}O_3$	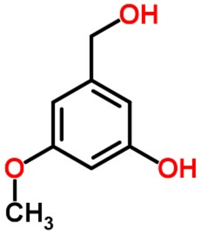	-	-	-	-	-	-

Supplementary Table 4. Continued.

Rt	Tentative identification	Kovat's Index	Lib Score	Formula of the molecule	Structure of the molecule	References					
						Antimicrobial Activity	Anti-diabetic activity	Anti-hemolysis activity	Anti-inflammatory activity	Anti-obesity activity	Cytotoxicity
32.457	2',5'-dimethyl crotonophenone	1462	86.6	$C_{12}H_{14}O$	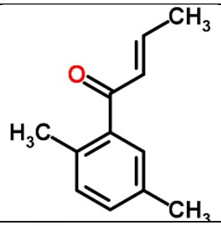	-	-	-	-	-	-
33.685	2,5,8-trimethyltetralin	1471	91.5	$C_{13}H_{18}$	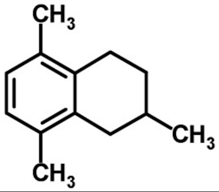	-	-	-	-	-	-
33.929	Dimethylmuconic acid	1499	85.7	$C_8H_{10}O_4$	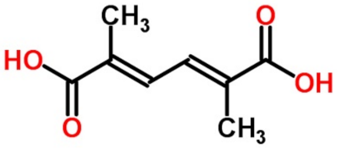	-	-	-	-	-	-
35.265	Cyclo(Gly-Pro)	1795	91.3	$C_7H_{10}N_2O_2$	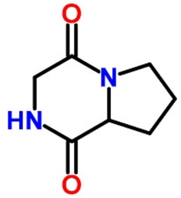	-	-	-	[63]	-	-
35.346	Cyclo(Leu-Pro)	1896	86.7	$C_{11}H_{18}N_2O_2$	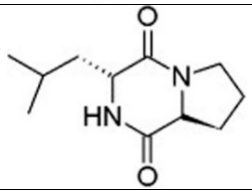	[5]	-	-	[6]	-	[7]

Supplementary Table 4. Continued.

Rt	Tentative identification	Kovat's Index	Lib Score	Formula of the molecule	Structure of the molecule	References					
						Antimicrobial Activity	Anti-diabetic activity	Anti-hemolysis activity	Anti-inflammatory activity	Anti-obesity activity	Cytotoxicity
36.475	Geranyl-α-terpinene	1968	88.7	$C_{20}H_{32}$	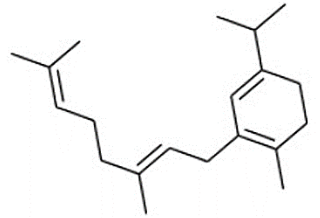	-	-	-	-	-	-
36.800	β-carboline	2009	93.0	$C_{11}H_8N_2$	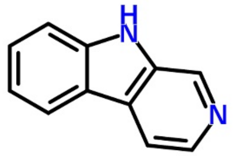	[64]	-	[64]	-	-	[64]
37.080	Cyclo(Phe-Pro)	2138	93.7	$C_{14}H_{16}N_2O_2$	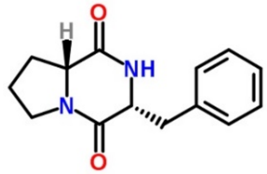	[38]	-	-	-	-	-

Supplementary Table 5. Genome mining of secondary metabolites of *Geotrichum candidum* and *Penicillium citrinum* isolates.

Penicillium citrinum isolate DSM 1997				
Cluster	Type	Size	Compound	Structure
1	T1pks	28,792	-	-
2	Nrps	18,748	-	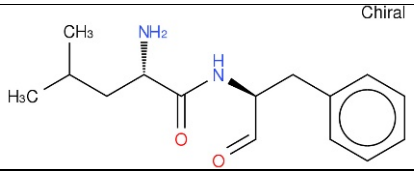
3	Other	42,474	-	-
4	Nrps	44,262	-	-
5	Other	36,943	-	-
6	T1pks	50,806	Equisetin biosynthetic gene cluster (18% of genes show similarity)	-
7	T1pks	59,370	Sorbicillin biosynthetic gene cluster (42% of genes show similarity)	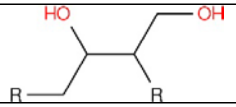
8	T1pks	28,328	-	-
9	T1pks-Nrps	51,926	-	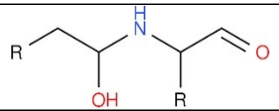
10	Nrps	41,023	-	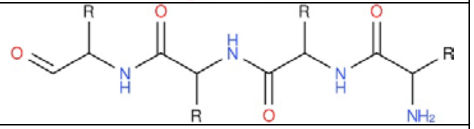
11	Other	39,102	-	-
12	Other	37,864	-	-
13	Terpene	21,274	-	-
14	Other	25,232	-	-
15	Other	34,917	-	-
16	T1pks	22,145	-	-

Supplementary Table 5. Continued.

Penicillium citrinum **isolate DSM 1997**				
Cluster	Type	Size	Compound	Structure
17	T1pks	47,260	Citrinin biosynthetic gene cluster (31% of genes show similarity)	-
18	Nrps	46,339	-	-
19	Nrps	85,967	-	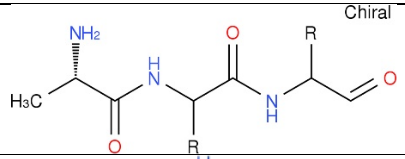
20	T1pks-Nrps	43,638	-	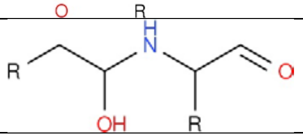
21	Other	38,012	-	-
22	Other	27,978	-	-
23	T1pks	46,584	-	-
24	Other	45,568	-	-
25	T1pks	33,688	Emericellin biosynthetic gene cluster (28% of genes show similarity)	-
26	T1pks	32,044	-	-
27	Indole	42,758	-	-
28	T1pks	48,262	-	-
29	T1pks	34,925	-	-

Supplementary Table 5. Continued.

Penicillium citrinum **isolate JCM 22607**				
Cluster	Type	Size	Compound	Structure
1	T1pks	45,584	Emericellin biosynthetic gene cluster (28% of genes show similarity)	-
2	T1pks	46,811	-	-
3	T1pks	48,261	-	-
4	Nrps	58,642	-	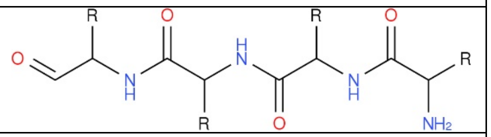
5	T1pks	57,346	Sorbicillin biosynthetic gene cluster (57% of genes show similarity)	-
6	T1pks	44,579	-	-
7	Nrps	45,914	-	-
8	Terpene	21,274	-	-
9	Other	44,893	-	-

Supplementary Table 5. Continued.

Penicillium citrinum **Core Genome**				
Cluster	Type	Size	Compound	Structure
1	T1pks	28,792	-	-
2	Nrps	18,748	-	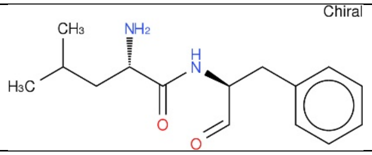
3	Other	42,474	-	-
4	Nrps	44,262	-	-
5	Other	36,943	-	-
6	T1pks	50,806	Equisetin biosynthetic gene cluster (18% of genes show similarity)	-
7	T1pks	59,370	Sorbicillin biosynthetic gene cluster (42% of genes show similarity)	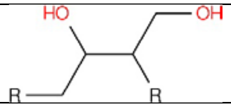
8	T1pks	28,327	-	-
9	T1pks-Nrps	39,576	-	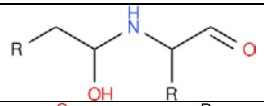
10	Nrps	41,023	-	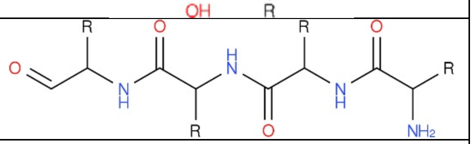
11	Other	39,102	-	-
12	Other	37,517	-	-
13	Terpene	21,274	-	-
14	Other	25,232	-	-
15	Other	29,211	-	-
16	T1pks	22,135	-	-

Supplementary Table 5. Continued.

Penicillium citrinum **Core Genome**				
Cluster	Type	Size	Compound	Structure
17	T1pks	47,260	Citrinin biosynthetic gene cluster (31% of genes show similarity)	-
18	Nrps	46,339	-	-
19	Nrps	85,967	-	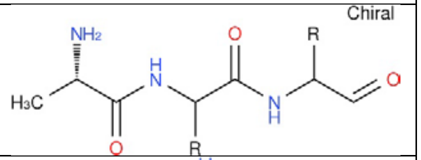
20	T1pks-Nrps	43,638	-	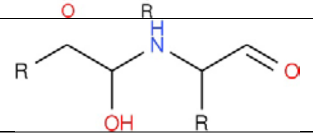
21	Other	38,012	-	-
22	Other	27,978	-	-
23	T1pks	46,584	-	-
24	Other	45,568	-	-
25	T1pks	33,688	Emericellin biosynthetic gene cluster (28% of genes show similarity)	-
26	T1pks	32,043	-	-
27	Indole	19,754	-	-
28	T1pks	48,262	-	-
Penicillium citrinum **isolate DSM 1997 Accessory genome**				
1	T1pks	18,735	-	-
Penicillium citrinum **isolate JCM 22607 Accessory genome**				
-	-	-	-	-

References

1. Bittner, S.; Scherzer, R.; Harlev, E. The five bromotryptophans. *Amino Acids* **2007**, 33, 19-42. DOI: 10.1007/s00726-006-0441-8.
2. Liu, H.; Jia, Y. (2017). Ergot alkaloids: synthetic approaches to lysergic acid and clavinet alkaloids. *Nat. Prod. Rep* **2017**, 34, 411-432. DOI: 10.1039/C6NP00110F.
3. Kongsaeree, P.; Prabpai, S.; Sriubolmas, N.; Vongvein, C.; Wiyakrutta, S. Antimalarial dihydroisocoumarins produced by *Geotrichum* sp., an endophytic fungus of *Crassocephalum crepidioides*. *J. Nat. Prod* **2003**, 66, 709-711.
4. Saeed, A.; Rafique, H.; Arshad, M. Total synthesis and antibacterial screening of (±)-7-butyl-6,8-dihydroxy-3-pentyl-3,4-dihydroisochromen-1-one. *J. Asian Nat. Prod. Res* **2013**, DOI: 10.1080/10286020.2013.817993.
5. Kumar, S.N.; Nath, V.S.; Chandran, R.P.; Nambisan, B. Cyclic dipeptides from rhabditid entomopathogenic nematodeassociated *Bacillus cereus* have antimicrobial activities. *World J. Microbiol. Biotechnol* **2014**, 30, 439-449. DOI: 10.1007/s11274-013-1461-7.
6. Lee, M-S.; Wang, S-W.; Wang, G-J.; Pang, K-L.; Lee, C-K.; Kuo, Y-H.; Cha, H-J.; Lin, R-K.; Lee, T-H. (2016). Angiogenesis inhibitors and anti-Inflammatory agents from *Phoma* sp. NTOU4195. *J. Nat. Prod* **2016**, 79, 2983-2990. DOI: 10.1021/acs.jnatprod.6b00407.
7. Mangamuri, U.K.; Muvva, V.; Poda, S.; Manavathi, B.; Bhujangarao, C.; Yenamandra, V. Chemical characterization and bioactivity of diketopiperazine derivates from the mangrove derived *Pseudonocardia endophytica*. *Egypt. J. Aquat. Res* **2016**, 42, 169-175.
8. Wattana-Amorn, P.; Charoenwongsa, W.; Williams, C.; Crump, M.P.; Apichaisataienchote, B. Antibacterial activity of cyclo(L-Pro-L-Tyr) and cyclo(D-Pro-L-Tyr) from *Streptomyces* sp. Strain 22-4 against phytopathogenic bacteria. *Nat. Prod. Res*. **2016**, 30, 1980-1983. DOI: 10.1080/14786419.2015.1095747.
9. Yang, M-L.; Kuo, P-C.; Hwang, T-L.; Wu, T-S. Anti-inflammatory Principles from *Cordyceps sinensis*. *J. Nat. Prod* **2011**, 74, 1996-2000. DOI: 10.1021/np100902f.
10. Capon, R.J.; Stewart, M.; Ratnayake, R.; Lacey, E.; Gill, J.H. Citromycetins and bilains A-C: new aromatic polyketides and diketopiperazines from Australian marine-derived and terrestrial *Penicillium* spp. *J. Nat. Prod* **2007**, 70, 1746-52. DOI: 10.1021/np0702483.
11. Gao, Y.; Liu, Q.; Liu, B.; Xie, C-L.; Cao, M-J.; Yang, X-W.; Liu, G-M. Inhibitory activities of compounds from the marine actinomycete *Williamsia* sp. MCCC 1A11233 variant on IgEmediated mast cells and passive cutaneous anaphylaxis. *J. Agric. Food Chem* **2017**, 65, 10749-10756. DOI: 10.1021/acs.jafc.7b04314.
12. Vazquez-Rivera, D.; Gonzalez, O.; Guzman-Rodriguez, J.; Diaz-Perez, A.L.; Ochoa-Zarzosa, A.; Lopez-Bucio, J.; Meza-Carmen, V.; Jesus Campos-Garcia, J. Cytotoxicity of cyclodipeptides from *Pseudomonas aeruginosa* PAO1 leads to apoptosis in human cancer cell lines. *BioMed Res. Int* **2015**, 197608. DOI: 10.1155/2015/197608.
13. Elsebai, M.F.; Saleem, M.; Tejesvi, M.V.; Kajula, M.; Mattila, S.; Mehiri, M.; Turpeinen, A.; Pirttila, M. Fungal phenalenones: chemistry, biology, biosynthesis and phylogeny. *Nat. Prod. Rep* **2014**, 31, 628. DOI: 10.1039/c3np70088g.
14. Wrigley, S.K.; Ainsworth, A.M.; Kau, D.A.; Martin, S.M.; Bahl, S.; Tang, J.S.; Hardick, D.J.; Rawlins, P.; Sadheghi, R.; Moore, M. Novel reduced benzo[j]fluranthen-3-ones from *Cladosporium* cf. *cladosporioides* with cytokine production and tyrosine kinase inhibitory properties. *J. Antibiot (Tokyo)* **2011**, 54, 479-88.
15. Fill, T.P.; dos Santos, R.M.G.; Barissonc, A.; Rodrigues-Filhoa, E.; Souza, A.Q.L. Co-production of bisphenylpropanoid amides and meroterpenes by an endophytic *Penicillium brasilianum* found in the root bark of *Melia azedarach*. *Z. Naturforsch* **2009**, 64 c, 355-360.

16. Guo, Z.; Ren, F.; Che, Y.; Gang Liu, G.; Liu, L. New bergamotane sesquiterpenoids from the plant endophytic fungus *Paraconiothyrium brasiliense. Molecules* **2015**, 20, 14611-14620. DOI: 10.3390/molecules200814611.
17. Lobermann, F.; Weisheit, L.; Dirk Trauner, D. Intramolecular Vinyl Quinone Diels–Alder Reactions: Asymmetric Entry to the Cordiachrome Core and Synthesis of (−) Isoglaziovianol. *Org. Lett* **2013**, 15, 4324–4326. DOI: 10.1021/ol401787n.
18. Dettrakul, S.; Surerum, S.; Shuleewan Rajviroongit, S.; Kittakoop, P. Biomimetic transformation and biological activities of globiferin, a terpenoid benzoquinone from *Cordia globifera*. *J. Nat. Prod* **2009**, 72, 861–865. DOI: 10.1021/np9000703.
19. Bodugam, M.; Javed, S.; Ganguly, A.; Torres, J.; Hanson, P.R. A pot-economical approach to the total synthesis of Sch-725674. *Org. Lett* **2016**, 18, 516–519. DOI: 10.1021/acs.orglett.5b03547.
20. Zhang, Y.; Zhu, T.; Fang, Y.; Liu, H.; Gu, Q.; Zhu, W. Carbonarones A and B, new bioactive g-pyrone and a-pyridone derivatives from the marine-derived fungus *Aspergillus carbonarius*. *J. Antibiot* **2007**, 60, 153-157.
21. Klykov, A.G.; Moiseenko, L.M.; Chaikinab, E.L.; Afiyatullovb, S.S.; Anisimov, M.M. Productivity and quality of Seeds of *Fagopyrum esculentum* Moench under treatment with virescenosides A and a sum of glycosides of marine Fungus *Acremonium striatisporum* at super low concentrations. *Russian Agr. Sci* **2013**, 39, 303–306.
22. Betina, V.; Micekova, D.; Nemec, P. Antimicrobial properties of cytochalasins and their alteration of fungal morphology. *J. Gen. Microbiol* **1972**, 71, 343-349.
23. Davis, J.A.; Sharma, S.; Mittra, S.; Sujatha, S.; Kanaujia, A.; Shukla, G.; Katiyar, C.; Lakshmi, B.S.; Bansal, V.S.; Bhatnagar, P.K. Antihyperglycemic effect of Annona squamosa hexane extract in type 2 diabetes animal model: PTP1B inhibition, a possible mechanism of action? *Indian J. Pharmacol* **2012**, 44, 326–332. DOI: 10.4103/0253-7613.96304.
24. Dutraa, F.F.; Alvesa, L.S.; Rodriguesa, D.; Fernandez, P.L.; de Oliveirac, R.B.; Golenbockc, D.T.; Zambonid, D.S.; Bozza, M.T. Hemolysis-induced lethality involves inflammasome activation by heme. *PNAS* **2014**, E4110–E4118. DOI: 10.1073/pnas.1405023111.
25. Jouda, J-B.; Tamokou, J-D.; Mbazoa, C.D.; Douala-Meli, C.; Sarkar, P.; Bag, P.K.; Wandji, J. Antibacterial and cytotoxic cytochalasins from the endophytic fungus *Phomopsis* sp. harbored in *Garcinia kola* (Heckel) nut. *BMC Complement. Altern. Med* **2016**, 16: 462. DOI: 10.1186/s12906-016-1454-9.
26. Lee, J.; Yi, J-M.; Kim, H.; Lee, Y.J.; Park, J-S.; Bang, O-S.; No Soo Kim, N-S. Cytochalasin H, an active anti-angiogenic constituent of the ethanol extract of *Gleditsia sinensis* Thorns. *Biol. Pharmacol. Bull* **2014**, 37, 6–12.
27. Graziano, T.S.; Cuzzullin, M.C.; Franco, G.C.; Schwartz-Filho, H.O.; deAndrade, E.D.; Groppo, F.C.; Cogo-Müller, K. Statins and antimicrobial effects: Simvastatin as a potential drug against *Staphylococcus aureus* biofilm. *PloS ONE* **2015**, 10(5): e0128098. DOI: 10.1371/journal.pone.0128098.
28. Alberts, A.W.; Chen, J.; Kuron, G.; Hunt, V.; Huff, J.; Hoffman, C.; Rothrock, J., et al. Mevinolin: A highly potent competitive inhibitor of hydroxymethylglutaryl-coenzyme a reductase and a cholesterol-lowering agent. *Proc. Natl. Acad. Sci. USA* **1980**, 77, 3957-3961.
29. Mahmoud, A.M.; Al-Abd, A.M.; Lightfoot, D.A.; Hany A.; El-Shemy, H.A. Anti-cancer characteristics of mevinolin against three different solid tumor cell lines was not solely p53-dependent. *J. Enzyme Inhib. Med. Chem* **2012**, 27, 673-679. DOI: 10.3109/14756366.2011.607446.
30. Liu, Y.N.; Xue, J.H.; Feng, N.; Wu, P.; Liu, X.Z.; Wei, X.Y. A new cyclodipeptide from the cultures of Geotrichum candidum. *Chin. Chem. Lett* **2007**, 18, 1081–1083. DOI: 10.1016/j.cclet.2007.07.003.
31. Woloshuk, C.P.; Sisler, H.D.; Dutky, S.R. Mode of action of the azasteroid antibiotic 15-Aza-24-Methylene-D-Homocholesta-8,14-Dien-3f8-ol in *Ustilago maydis*. *Antimicrob. Agents Chemother* **1979**, 16, 98-103.

32. https://www.google.ch/patents/US4008238
33. https://www.google.ch/patents/US40082
34. https://www.google.ch/patents/US40082
35. Takahata, H.; Momose, T. Simple indolizidine alkaloids In: The alkaloids: chemistry and pharmacology. 44, 189-256. Academic Press, **1993**, The United States. pp. 245.
36. Manzeera, A.S.; Sireesha, P.N.; Aswini, M.; Rao, P.M.; Sree, K.N.; Mallikharjunarao, K.L.N. Synthesis and anti-inflammatory activity of pyrazine derivatives. *Asian J. Res. Pharmacol. Sci. Biotec* **2013**, 1, 10-15.
37. Kumar, M.S.; Pal, A.K. Investigation of bioactivity of extracts of Marine Sponge, *Spongosorites halichondrioides* (Dendy, 1905) from western coastal areas of India. *Asian Pac. J. Trop. Biomed* **2012**, S1784-S1789.
38. Strom, K.; Sjogren, J.; Broberg, A.; Schnurer, J. *Lactobacillus plantarum* MiLAB 393 produces the antifungal cyclic dipeptides cyclo(L-Phe–L-Pro) and cyclo(L-Phe–trans-4-OH-L-Pro) and 3-Phenyllactic acid. *Appl. Environ. Microbiol* **2002**, 68, 4322-4327. DOI: 10.1128/AEM.68.9.4322–4327.2002.
39. Kumarihamy, M.; Khan, S.I.; Jacob, M.; Tekwani, B.L.; Duke, S.O.; Ferreira, D.; Nanayakkara, N.P.D. Antiprotozoal and antimicrobial compounds from the plant pathogen *Septoria pistaciarum*. *J. Nat. Prod* **2012**, 75, 883–889. DOI: 10.1021/np200940b.
40. Alshaibani, M.M.; MohamadZin, N.; Jalil, J.; Sidik, N.M.; Ahmad, S.J.; Kamal, N.; Edrada-Ebel, R. Isolation, purification, and characterization of five active diketopiperazine derivatives from endophytic *Streptomyces* SUK 25 with antimicrobial and cytotoxic activities. *J. Microbiol. Biotechnol* **2017**, 27, 1249-1256. DOI: 10.4014/jmb.1608.08032.
41. Abdel-Lateff, A. Chaetominedione, a new tyrosine kinase inhibitor isolated from the algicolous marine fungus *Chaetomium* sp. *Tetrahedron Lett* **2008**, 49, 6398–6400. DOI: 10.1016/j.tetlet.2008.08.064.
42. https://openi.nlm.nih.gov/detailedresult.php?img=PMC4483665_marinedrugs-13-03950-g018&req=4
43. Gianetti, B.M.; Steghlich, W.; Quack, W.; Anke, T.; Oberwinkler, F. Antibiotics from basidiomycetes, VI. Merulinic acids A, B, and C, new antibiotics from *Merulinus tremellosus* and *Phlebia radiata*. *Z. Naturforsch C* **1978**, 33(11-12), 807-816.
44. http://www.genome.jp/dbget-bin/www_bget?dr:D02899
45. https://en.wikipedia.org/wiki/Citrinin
46. Johannessen, L.N.A.M.; Nilsen, A.M.; Lovik, M. The mycotoxins citrinin and gliotoxin differentially affect production of the pro-inflammatory cytokines tumour necrosis factor-a and interleukin-6, and the anti inflammatory cytokine interleukin-10. *Clin. Exp. Allergy* **2005**, 35, 782–789. DOI: 10.1111/j.1365-2222.2005.02249.x.
47. Andrade, R.; Ayer, W.A.; Trifonov, L.S. The metabolites of *Trichoderma longibrachiatum*. III. Two new tetronic acids: 5-Hydroxyvertinolide and bislongiquinolide. *Aust. J. Chem* **1997**, 50, 255-257.
48. Derntl, C.; Guzman-Chavez, F.; Mello-de-Sousa, T.M.; Busse, H-J.; Driessen, A.J.M.; Mach, R.L.; Mach-Aigner, A.R. *In vivo* study of the sorbicillinoid gene cluster in *Trichoderma reesei*. *Front. Microbiol* **2017**, 8:2037. DOI: 10.3389/fmicb.2017.02037.
49. Nissen, L.; Zatta, A.; Stefanini, I.; Grandi, S.; Sgorbati, B.; Biavati, B.; et al. Characterization and antimicrobial activity of essential oils of industrial hemp varieties (*Cannabis sativa* L.). *Fitoterapia* **2010**, 81, 413–419. DOI: 10.1016/j.fitote.2009.11.010.
50. Russo, E.B. Taming THC: potential cannabis synergy and phytocannabinoid-terpenoid entourage effects. *Brit. J. Pharmcol* **2011**, 163, 1344–1364. DOI: 10.1111/j.1476-5381.2011.01238.x.
51. Hao, D.; Xiao, J.G.; Pei G.X. Medicinal plants: Chemistry, biology and omics. Elsevier Science, **2015**, pp. 694.

52. Marei, G.I.K.H.; Rasoul, M.A.A.; Abdelgaleil, S.A.M. Comparative antifungal activities and biochemical effects of monoterpenes on plant pathogenic fungi. *Pest Biochem. Physiol* **2012**, 103, 56-61.
53. Quintans-Júnior, L.; Moreira, J.C.F.; Pasquali, M.A.B.; Rabie, S.M.S., et al. Antinociceptive activity and redox Profile of the Monoterpenes(+)-Camphene, p-Cymene, and Geranyl Acetate in Experimental Models. *ISRN Toxicol* **2013**, 459530. DOI: 10.1155/2013/459530.
54. Vallianou, I.; Peroulis, N.; Pantazis, P.; Hadzopoulou-Cladaras, M. Camphene, a plant-derived monoterpene, reduces plasma cholesterol and triglycerides in hyperlipidemic rats independently of HMG-CoA reductase activity. *PLoS ONE* **2011**, 6: e20516. DOI: 10.1371/journal.pone.0020516.
55. Lingappa, B.T.; Prasad, M.; Lingappa, Y.; Hunt, D.F.; Biemann, K. Phenethyl alcohol and tryptophol: Autoantibiotics produced by the fungus *Candida albicans*. *Science* **1969**, 163 (3863), 192-194. DOI: 10.1126/science.163.3863.192.
56. Nakahara, K.; Alzoreky, N.S.; Yoshihashi, T.; Nguyen, H.T.T.; Trakoontivakorn, G. Chemical composition and antifungal activity of essential oil from Cymbopogon nardus (Citronella grass). *JARQ* **2003**, 37, 249-252.
57. Rajapakse, N.W.; De Miguel, C.; Das, S.; Mattson, D.L. Exogenous L-arginine ameliorates angiotensin II-induced hypertension and renal damage in rats.. *Hypertension* **2008**, 52, 1084-1090.
58. Frezza, C.; Mauro, C. Editorial: The Metabolic Challenges of Immune Cells in Health and Disease. *Front. Immunol* **2015**, 6: 293. DOI: 10.3389/fimmu.2015.00293.
59. Hammer, K.A.; Carson, C.F.; Riley, T.V. Effects of *Melaleuca alternifolia* (Tea Tree) essential oil and the major monoterpene component Terpinen-4-ol on the development of single- and multistep antibiotic resistance and antimicrobial susceptibility. *Antimicrob. Agents Chemother* **2012**, 56, 909-915. DOI: 10.1128/AAC.05741-11.
60. Hart, P.H.; Brand, C.; Carson, C.F.; Riley, T.V.; Prager, R.H.; Finlay-Jones, J. J. Terpinen-4-ol, the main component of the essential oil of *Melaleuca alternifolia* (tea tree oil), suppresses inflammatory mediator production by activated human monocytes. *Inflamm. Res* **2000**, 49, 619–626.
61. Ebada, M.E. Cuminaldehyde: A potential drug candidate. *J. Pharmacol. Clin. Res* **2017**, 2, 555585. DOI: 10.19080/ JPCR.2017.02.555585002.
62. Gille, L.; Monzote, L.; Stamberg, W.; Staniek, K. Toxicity of ascaridole from *Chenopodium ambrosioides* in mammalian mitochondria. *BMC Pharmacol* **2010**, 10 (Suppl 1): A10. DOI: 10.1186/1471-2210-10-S1-A10.
63. Ferro, J.N.D.S.; de Aquino, F.L.T.; de Brito, R.G.; Santos, P.L.D. et al. Cyclo-Gly-Pro, a cyclic dipeptide, attenuates nociceptive behaviour and inflammatory response in mice. *Clin. Exp. Pharmacol. Physiol* **2015**, 42, 1287–129. DOI: 10.1111/1440-1681.12480.
64. Cao, R.; Peng, W.; Wang, Z.; Xu, Anlong. β-Carboline Alkaloids: Biochemical and Pharmacological Functions. *Curr. Med. Chem* **2007**, 14, 479-500.
